# A high intensity vs. casual speed walking intervention to reverse frailty among older adults: a cluster randomized controlled trial

**DOI:** 10.1371/journal.pone.0357605

**Published:** 2026-09-30

**Authors:** Margaret Danilovich, Daniel S. Rubin, David E. Conroy, Laura Diaz, Ying Cheung, Daniel Corcos, Lauren Balmert Bonner

**Affiliations:** 1 Department of Physical Therapy and Human Movement Sciences, Northwestern University, Feinberg School of Medicine, Chicago, Illinois, United States of America; 2 Department of Anesthesia and Critical Care, University of Chicago, Chicago, Illinois, United States of America; 3 School of Kinesiology, University of Michigan, Ann Arbor, Michigan, United States of America; 4 Department of Obstetrics and Gynecology, Northwestern University Feinberg School of Medicine, Chicago, Illinois, United States of America; 5 Department of Preventive Medicine (Biostatistics), Northwestern University Feinberg School of Medicine, Chicago, Illinois, United States of America; 6 Department of Physical Therapy & Human Movement Sciences and McCormick School of Engineering, Northwestern University, Chicago, Illinois, United States of America; Japanese Academy of Health and Practice, JAPAN

## Abstract

Interventions to reduce frailty and promote healthy aging are critical to improving quality of life and reducing disability in older adults. Walking is the most common form of exercise among older adults; however, the optimal walking intensity for prefrail and frail adults remains unclear. We conducted a cluster-randomized, assessor- and participant-blinded trial to determine whether high-intensity walking (HIW) improves frailty more than casual-speed walking (CSW). Fourteen retirement communities were randomized to either HIW or CSW using a modified minimal sufficient balance randomization scheme. We recruited 165 prefrail and frail adults aged ≥60 years, of whom 105 were included in the analytic cohort. Participants were able to ambulate at least 10 feet with moderate assistance or less. The HIW group targeted 70% of heart rate maximum (HRmax), while the CSW group walked at self-selected speeds below 60% HRmax. Supervised walking sessions were 45 minutes, three times per week, for 16 weeks. The primary outcome was improvement by at least one frailty category on the SHARE-FI (Survey of Health Among Retired Europeans – Frailty Index). Secondary outcomes included the Timed Up and Go, Short Physical Performance Battery, Berg Balance Scale, and 6-Minute Walk Test (6MWT), measured at baseline and 16 weeks. Participants had a mean (SD) age of 79.2 (8.0) years; 79% were female, and 75% were non-Hispanic White. Improvement in frailty category occurred in 32 of 46 (69.6%) HIW participants versus 26 of 56 (46.4%) CSW participants (OR = 2.91, 95% CI 1.19–7.13, p = 0.02). HIW participants showed greater gains in gait speed (mean difference = 0.08 m/s, 95% CI 0.03–0.13, p = 0.036) and 6MWT distance (mean difference = 48.99 m, 95% CI 21.1–76.9, p = 0.0088). Mean HRmax was 71% in HIW vs. 59% in CSW (p < 0.001). Median completed sessions were similar (HIW = 35; CSW = 36; p = 0.07). This cluster-randomized trial demonstrates that high-intensity walking can reverse frailty more effectively than casual-speed walking due to increased physiologic stimuli during more intensive activity. This study is registered at ClinicalTrials.gov ID NCT03654807 (https://clinicaltrials.gov/study/NCT03709251) Registered 10/12/2018.

## Introduction

Frailty is a geriatric syndrome in which the phenotype criteria are weight loss, fatigue, weakness, slow walking speed, and low physical activity. Physically frail individuals have three of the five criteria, while pre-frail individuals have one or two [1]. Frailty is associated with a wide array of adverse health outcomes including higher health expenditures, increased risk for hospitalization, and all-cause mortality [1]. With an increasingly aging global population, reducing and reversing frailty is a critical societal priority.

Physical activity (PA) has multi-system health benefits for older adults and is the most recommended frailty management intervention [2]. To date, there is strong evidence that exercise interventions can prevent or delay frailty progression [3]. Yet, there remains no clear evidence that existing frailty can be reversed through exercise interventions and there are no clear guidelines regarding the most effective exercise parameters to achieve this aim [3].

Walking is the most preferred PA mode for older adults [4] and increasing daily step count may reduce the presence of frailty. Observational research finds a dose-response relationship with higher daily step counts associated with lower frailty prevalence [5]. This relationship persists even among those with very low step counts (less than 3,000 per day) suggesting any increase in step counts may reduce frailty presence. Step counts can be increased by walking for longer durations or walking faster for the same duration. Observed as the 6^th^ vital sign, gait speed is a key frailty indicator, as slow gait speed is one of the most frequently observed and prevalent criteria of frailty [6]. Since gait speed predicts mortality, falls, loss of independence, and cognitive decline [7], and is an important clinical metric of overall health [8], interventions which improve capacity to ambulate faster and over longer distances (e.g., a greater volume of activity) may be particularly therapeutic for those with frailty.

While walking interventions improve functional capacity, their effectiveness in modifying the frailty phenotype remains unclear. Walking should influence at least two frailty criteria: walking speed and physical activity. In the LIFE-P study [9], older adults who walked 150 minutes each week at a low to moderate intensity had a 0.43 out of 5 decrease in the number of frailty criteria present after 6 months of intervention. Interestingly, when physical activity was omitted from the frailty calculation, the intervention did not impact frailty, demonstrating that low-moderate intensity walking had null effects on the frailty syndrome while predominantly influencing physical activity. Thus, improvements in frailty after exercise training may be driven by changes in specific frailty criteria (e.g., physical activity) and whether these changes are potent enough to reverse the frailty syndrome remains unclear.

The primary aim of this study was to test the effect of walking intensity on frailty category at the participant level by manipulating walking speed. Those in the casual speed walking arm walked at a casual, relaxed speed targeted to be less than 60% of estimated maximum heart rate (HR_max_) with an RPE below 13 or a moderate intensity level. Those in the high intensity walking arm walked fast with a goal for greater than 70% HR_max_ or greater than 15 RPE or a vigorous intensity level. The rationale for these intensity levels was to test two distinct intensity levels: moderate vs. vigorous [10]. As such, our study design did not include a no-treatment control group as we were comparing active exercise strategies to determine which was more effective in reducing frailty. We defined improvement as a binary indicator of reduction by at least one frailty category on the SHARE-FI from baseline to follow-up. We hypothesized that the higher intensity intervention arm would have a greater percentage of participants with frailty reduction and improvement compared to the casual speed walking arm. The secondary aim was to test the effect of walking intensity on a battery of physical performance measures and patient-reported outcomes: SHARE-FI continuous score, mobility (gait speed, 6 Minute Walk Test, Timed Up and Go), physical function (Short Physical Performance Battery, PROMIS-Global Health), balance (Berg Balance Scale, Falls Efficacy Scale), and physical activity (PA) volume.

## Methods

### Study design

We conducted a double-masked, cluster-randomized controlled intervention trial in independent living retirement communities to investigate the effects of walking intensity on frailty category (Aim 1), and frailty continuous score, mobility, physical functioning, balance, and PA assessed during a one-week ambulatory monitoring period before and after the 16-week intervention (Aim 2). We used a cluster randomization approach to prevent contamination and keep participants blinded to group assignment by avoiding both potential and enrolled participants from observing treatment sessions of different intensities given that intervention sessions were held on-site within the retirement community in public hallways. The Northwestern University institutional review board approved the study protocol. All participants provided written informed consent after the retirement community was randomized, but prior to any intervention, and data was collected and stored through Northwestern’s REDCap. The Consort diagram is shown in Fig 1 and the CONSORT checklist can be seen in S1 Checklist with the trial protocol found in S2 Protocol. The study is registered at: ClinicalTrials.gov ID NCT03654807 https://clinicaltrials.gov/study/NCT03709251.

**Fig 1:**
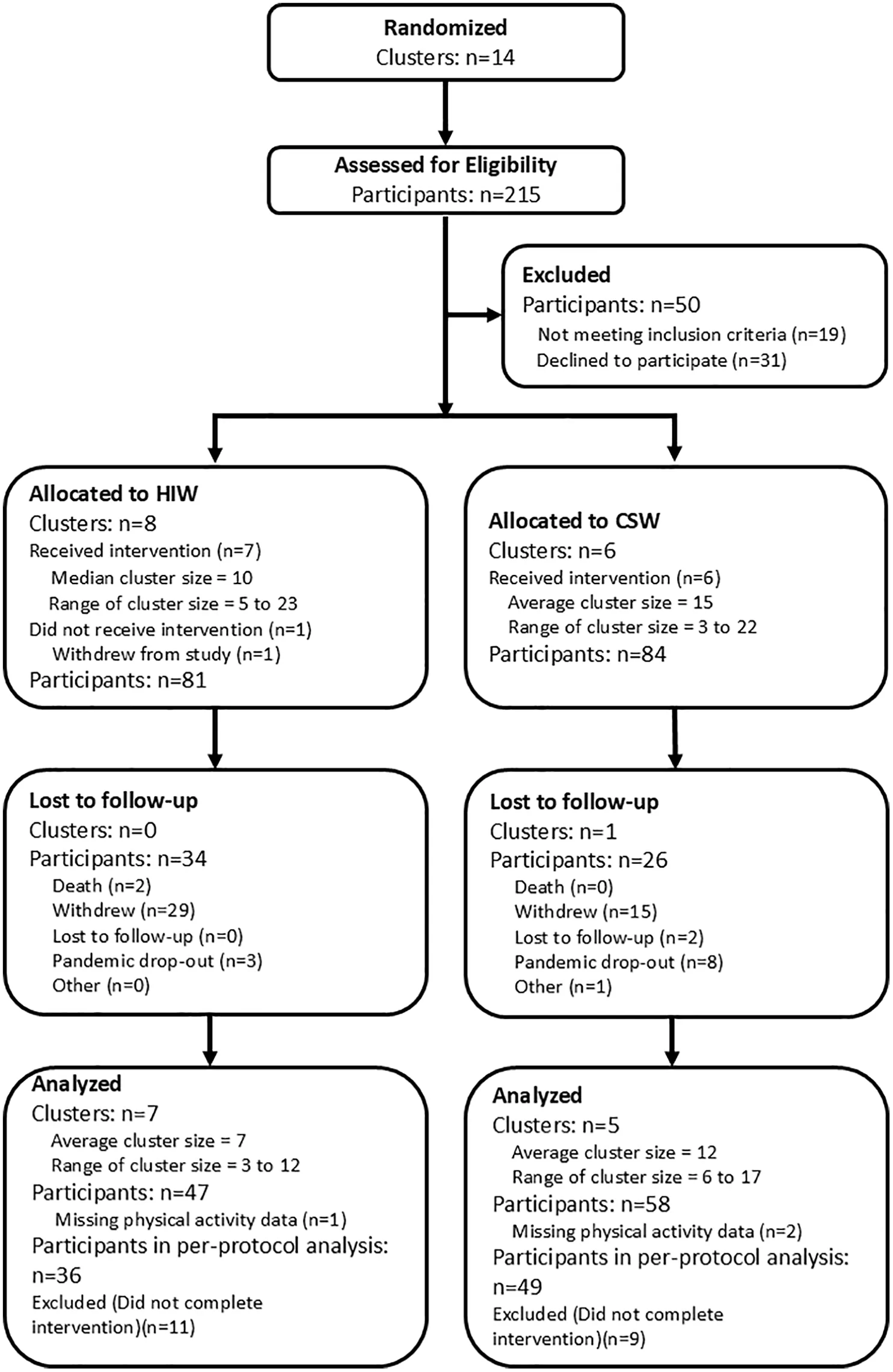
Consort diagram.

### Study Participants

Retirement communities were eligible if they were willing to present this study to their residents and provide space in their community to hold intervention sessions. We recruited communities through personal networks and professional organizations to ensure a diverse sample of communities based on rental rates/socioeconomic status, race/ethnicity of residents, and geographic area (urban/suburban). Participants with pre-frailty or frailty as determined by the SHARE-FI were enrolled in the study. Participants were >60 years of age, able to ambulate greater than 10 feet with moderate physical assistance or less with or without an assistive device and permanent residents of one of the enrolled independent living communities. Participants were excluded if they had uncontrolled cardiovascular, metabolic, renal, or respiratory disease that limited exercise participation.

### Recruitment, screening, and baseline testing

Recruitment occurred via flyers and presentations at the enrolled retirement communities. Potential participants were screened using the Exercise Assessment and Screening for You [11] to determine if physician clearance for participation was needed. Participants who met inclusion criteria underwent baseline assessments administered by a licensed physical therapist. All assessments occurred on site at the retirement community utilizing standardized equipment to ensure consistency between sites. Recruitment began November 1st 2018, was paused due to the COVID-19 pandemic from March 2020 to April 2021 and concluded in July 30th 2022.

### Randomization and masking

We randomized 14 independent living retirement communities to either a: 1) high intensity (HIW) or 2) casual speed (CSW) walking intervention. Retirement communities were randomized via a modified minimal sufficient balance randomization scheme for site level randomization by the unblinded statistical team (S2 Table in S3 File) [12]. Both the outcomes assessor and participants were blinded to group assignment. Since randomization occurred at the site level, participants were unaware of the other treatment arm as the consent materials indicated enrollment in a walking intervention with no mention of different walking intensities.

### Intervention

All participants were scheduled for three sessions per week for the 16-week intervention which was delivered via one-on-one sessions led by a research assistant. All research assistants received approximately two weeks of training by the study PI in monitoring vitals and guarding participants during ambulation. All intervention sessions occurred within the hallways and stairwells at the participant’s retirement community. The intervention consisted of three phases: Phase 1 – acclimation (Week 1, sessions 1–3), Phase 2 – ramp-up (Weeks 2–4, sessions 4–12), and Phase 3 – intervention (Weeks 5–16, sessions 13–48). The intention of a ramp up was to provide orientation to exercise prior to beginning the three-month intervention which was chosen as a minimum amount of time to see treatment effects. Details of each phase and walking activities are found in S3 Table and S2 Table in S3 File. HIW participants aimed to achieve >70% of heart rate maximum (HR_max_) or >15 on the Rating of Perceived Exertion (RPE) to facilitate high intensity while CSW participants walked at < 50% of HR_max_ or < 13 RPE. Given the impact of beta-blocker medications on heart rate and the high prevalence of beta-blocker use in our sample (72%), we used both HR and RPE to measure intensity [13].

Prior to beginning the intervention, we estimated participant’s HR_max_ using the Tanaka formula: Age-predicted HR_max_ = 208–0.7 x age [14] and used the Karvonen formula [15]. [Target HR = (HR_max_-HR_rest)_ x %intensity) + HR_rest_] to calculate each participant’s target HR thresholds for 70% or 50% of HR_max_ depending on group assignment. Research assistants monitored heart rate throughout sessions using a Nonin pulse oximeter and provided standardized feedback to participants in both groups following a standardized script (S3 Table in S3 File). The only difference in intervention sessions between groups was intensity of the session which was achieved via manipulating walking speed.

### Primary outcome

The primary outcome was a binary indicator of improvement by at least one frailty category defined by the SHARE-FI from baseline to 4 months. A binary indicator was used to distinguish individuals who experienced clinically meaningful improvement from those with stable or worsening frailty status. The SHARE-FI is based on a modified Fried frailty phenotypic, assesses frailty criteria through a freely accessible web-based calculator, [16] and evaluates frailty based on a measure of grip strength using a handgrip dynamometer and four self-reported items: fatigue, loss of appetite and/or eating less than usual, difficulties climbing stairs and/or walking 100 meters, and frequency of physical activity which makes it feasible and efficient for frailty administration in community settings. Both a continuous and categoric score (e.g., frail, pre-frail, or non-frail) are provided. The SHARE-FI is intended for community-dwelling adults aged 50 and over and has been shown to be a valid and reliable measure of frailty [16]. For females, SHARE-FI scores < 0.315 are considered non-frail, scores from 0.316 to 2.130 are pre-frail, and frail scores are values > 2.131. For males, scores < 1.211 indicate non-frail, scores from 1.212 to 3.005 are pre-frail, and frail scores are values > 3.006.

### Secondary outcomes

Secondary outcomes included change in the SHARE-FI continuous score and a battery of physical performance and patient-reported outcome measures as outlined below.

#### Mobility.

Gait speed was measured over 10 meters at self-selected (“walk at your normal comfortable pace”) and fastest-possible speeds (“as fast as you safely can”) with two trials averaged for analysis. The 6-Minute Walk Test was conducted in a retirement community hallways using standardized instructions and recording total meters walked. The Timed Up and Go (TUG) was performed using a standardized chair height at the participant’s self-selected usual speed.

#### Physical function.

We assessed physical functioning using the Short Physical Performance Balance (SPPB). The SPPB comprises gait speed, static balance, and sit to stand (chair rise) movements to provide an overall assessment of functional status and physical performance [17]. We also administered the PROMIS-Global Health questionnaire; a self-reported measure of global ratings across five domains (physical function, fatigue, pain, emotional distress, and social health) [18].

#### Balance.

To assess balance, we used the Berg Balance Scale and Falls Efficacy Scale. The Berg is a valid and reliable 14-item tool to evaluate static and dynamic balance among older adults. Scores on the Berg range from 0–56 with scores of 0–20 indicating high fall risk, scores of 21–40 indicating medium fall risk, and scores of 41–56 indicating low fall risk [19]. We administered the Falls Efficacy Scale questionnaire, a widely-used measure among older adults that evaluates the fear of falling and balance confidence. In this 10-item questionnaire with scores ranging from 0 to 100, participants rated their confidence in performing a variety of tasks using a 10-point Likert Scale. Scores of 70–79 indicate a fear of falling while scores of 80–100 indicate a fall risk [20].

#### Physical Activity.

We used activPAL activity monitors placed on the thigh and worn for a one-week ambulatory monitoring period in the week preceding intervention start and immediately following intervention conclusion. The activPAL is a small, lightweight triaxial accelerometer that samples data at 20 Hz to record both durations and frequencies of walking, standing, sitting, and lying events. Data were processed using PAL Batch Software Suite version 7 [21]. We analyzed activPAL accelerometer data for those with at least 4 days of valid wear days for both baseline and follow-up to calculate reliable person-level estimates at each measurement occasion. A valid day was defined as a day with at least 10 waking hours of wear time [22]. We calculated average daily step count (on valid days) to represent physical activity volume.

#### Additional outcomes.

We calculated each participant’s average percentage of maximum heart rate across tasks excluding warm-up and cool-down (measured heart rate divided by estimated maximum heart rate for each task and then averaged across the seven tasks in each session) to calculate average exercise intensity. Adherence was determined by the number the exercise sessions completed out of a possible 48 sessions. The study developed guidelines for adherence which defined a completed session as one in which a participant completed at least four out of seven walking tasks. Participants who completed more than 50% of tasks (4/7) tasks per session for at least 80% of Phase 3 intervention sessions (29/36 sessions) were considered to have “completed” the 16-week intervention and were considered in the per-protocol analysis. Adverse events were monitored during each intervention session and as reported by participants throughout the 16-week intervention.

### Statistical analysis

Descriptive statistics were used to summarize participant characteristics, as well as session-level data to characterize adherence to the protocol and dose of the intervention as indicators of treatment fidelity. The RPE and HR values were averaged across all tasks within a session (excluding the warm-up and cool-down) for each participant and then averaged across all intervention phase sessions.

Analyses were based on a modified intention-to-treat (ITT) principle, whereby all randomized participants with post-intervention data on at least one outcome measure were included in the analysis, regardless of number of completed sessions. Data from two participants from the CSW arm and one in the HIW arm did not have follow-up SHARE-FI data due to REDCap data loss which is why n = 102 were analyzed. Sensitivity analyses were conducted using the per-protocol sample.

The primary analysis used a generalized linear mixed model to estimate the odds of improvement in frailty category at 16 weeks in the HIW arm compared to the CSW arm. The model included fixed effects for arm and baseline SHARE-FI score. A random site effect accounted for clustering of participants within a retirement center. Linear mixed models were employed for secondary outcomes with fixed arm and baseline scores, and a random cluster effect. A similar model with random participant effect was used to compare changes in physical activity data over time. A pre-specified gatekeeping approach controlled for multiple testing of primary and secondary outcome families. Specifically, the primary outcome was tested at a 5% level of significance, and the secondary outcome family was tested at an overall 5% level of significance only following a significant primary outcome. The Hochberg approach was employed within the secondary outcome family to account for multiple testing. Sensitivity analyses also considered imputations for missing data.

### Sample size considerations

A priori sample size calculations were based on detecting differences in the proportion of improvement in SHARE-FI category between arms. We anticipated the CSW intervention arm to exhibit small improvements in frailty category, ranging from 1%−10%. We anticipated an approximate 25%−50% improvement in the HIW arm, corresponding to a difference of 20%−45% between arms. With limited knowledge on intra-cluster correlation, power considerations allowed for varying levels of correlation within cluster. Target sample sizes of 16 participants per site with 8 sites total, for a total of 128 participants, provided adequate power to detect these differences under a range of assumptions. Sample sizes were inflated to assume 20% attrition at both the site and participant level for enrolling 10 retirement communities with 20 participants each. Our final analytic sample size (assuming 7 CSW clusters with an average cluster size of 7 and 5 HIW clusters with an average cluster size of 12) provided 90% power to detect differences in proportions of 17%−30%.

## Results

### Retirement communities

Fourteen independent living retirement communities were enrolled and randomized (n = 8 in HIW and n = 6 in CSW). One community did not allow participant recruitment after randomization and was withdrawn. Estimates of the minority resident population ranged from 0% to 100% (median = 11.7%; IQR 1%, 63%) and cognitive impairment ranged from 0% to 30% (median = 6.3%; IQR 1.3%, 10%) based on self-reported data from retirement community administrators. Communities ranged in size from 100 to 600 residents, were in urban and suburban areas throughout the Chicagoland, Illinois, USA metropolitan area, and varied in rent rates ranging from $1,300 to over $8,000 USD per month rent.

### Baseline characteristics of participants

We screened 215 participants for eligibility from 13 randomized retirement communities; 165 (77%) were enrolled and 105 (49%) completed the intervention and were included in the analytic cohort: n = 47 in HIW and n = 58 in CSW (mean [SD] age, 79.2 [8.05]; age range 62–96 years; 83 [79%] were female; 79 [75%] were non-Hispanic White and 65% used an assistive device when ambulating (Table 1). Of note, n = 34 in HIW and n = 26 in CSW were not included in the analytic cohort due to withdrawal, lost-to-follow-up, or death (Fig 1). At baseline, 41% of participants were frail and 59% were pre-frail. Groups were well matched across demographic characteristics (Table 1). Table 4 in S3 File shows the participant demographics and baseline outcome measures between included and excluded participants. Participants who were excluded due to not completing follow-up measures were slightly older and had worse physical function.

**Table 1:** Participant Demographics.

Characteristic	Overall, N = 105^1^	CSW, N = 58^1^	HIW, N = 47^1^
**Age**			
Mean (SD)	79·2 (8·0)	79·3 (7·8)	79·2 (8·4)
**Sex**			
Female	83 (79%)	41 (71%)	42 (89%)
Male	22 (21%)	17 (29%)	5 (11%)
**Race**			
African-American	22 (21%)	13 (22%)	9 (19%)
Asian	2 (1·9%)	1 (1·7%)	1 (2·1%)
Non-White Hispanic	1 (1·0%)	0 (0%)	1 (2·1%)
Other	1 (1·0%)	1 (1·7%)	0 (0%)
White	79 (75%)	43 (74%)	36 (77%)
**Assistive Device**			
Cane	29 (28%)	18 (31%)	11 (23%)
None	37 (35%)	21 (36%)	16 (34%)
Other	4 (3·8%)	4 (6·9%)	0 (0%)
Walker	35 (33%)	15 (26%)	20 (43%)
**High Blood Pressure**	76 (72%)	43 (74%)	33 (70%)
**Diabetes**	24 (23%)	14 (24%)	10 (21%)
**Hip Pain**	37 (35%)	20 (34%)	17 (36%)
**Knee Pain**	47 (45%)	24 (41%)	23 (49%)
**Ankle Pain**	24 (23%)	8 (14%)	16 (34%)

^1^n (%)

### Primary outcomes

In the HIW group, 69.6% of participants had an improvement in SHARE-FI category compared to 46.4% of CSW participants (Table 2 and Fig 2A). We found significantly increased odds of improving at least one SHARE-FI frailty category in the HIW group (OR=2.91 [reference group = CSW], 95% CI 1.19, 7.13, p = 0.02, N = 102). The per-protocol analysis yielded consistent results, identifying a significant increase in odds of improving by at least one frailty category (OR = 3.41 [reference group = CSW], 95% CI 1.32, 8.84, p = 0.01).

**Table 2:** Primary and Secondary Outcome Modeling Results.

					Model Results			
Outcome	Time		CSW	HIW	N^1.^	OR/ Mean Difference (95% CI)	P-value^2.^	ICC^3.^
Frailty Category N (%)	Baseline	Pre-Frail	35 (60·3)	27 (57·4)				
		Frail	23 (39·7)	20 (42·6)				
	Follow-up	Non-Frail	15 (26·8)	23 (50·0)				
		Pre-Frail	28 (50·0)	17 (37·0)				
		Frail	13 (23·2)	6 (13·0)				
	Change from Baseline	Improved	26 (46·4)	32 (69·6)	102	2·91, (1·19, 7·13)	0·0200	0·014
Frailty Score	Baseline		2·35 (0·97)	2·10 (1·10)				
	Follow-up		1·20 (1·47)	0·61 (1·29)				
	Change from Baseline		−1·19 (1·66)	−1·51 (1·18)	102	−0·5, (−1·12, 0·12)	0·6636	0·039
Gait Speed – Usual	Baseline		0·81 (0·21)	0·80 (0·23)				
	Follow-up		0·82 (0·20)	0·89 (0·23)				
	Change from Baseline		0·01 (0·12)	0·10 (0·17)	102	0·08, (0·03, 0·13)	0·0360	0·000
Gait Speed – Fast	Baseline		1·01 (0·27)	1·03 (0·33)				
	Follow-up		1·07 (0·28)	1·15 (0·32)				
	Change from Baseline		0·05 (0·18)	0·12 (0·21)	102	0·07, (0, 0·15)	0·4088	0·001
6 Minute Walk Test (meters)	Baseline		252·28 (82·97)	262·14 (95·12)				
	Follow-up		264·07 (97·40)	317·48 (100·34)				
	Change from Baseline		10·26 (69·01)	57·97 (56·86)	102	48·99, (21·1, 76·87)	0·0088	0·028
Timed Up and Go (seconds)	Baseline		16·41 [13·17, 20·09]	16·56 [12·88, 19·12]				
	Follow-up		14·67 [12·85, 18·55]	13·85 [11·60, 17·09]				
	Change from Baseline		−0·37 [−2·50, 1·82]	−1·50 [−3·88, −0·51]	102	−0·92, (−3·15, 1·3)	0·8642	0·006
SPPB	Baseline		6·66 (2·69)	6·74 (2·66)				
	Follow-up		7·41 (2·63)	7·74 (2·82)				
	Change from Baseline		0·66 (1·44)	1·02 (2·15)	102	0·35, (−0·32, 1·03)	0·8642	0·000
Berg Balance Scale	Baseline		42·00 [37·00, 48·00]	42·00 [34·00, 49·50]				
	Follow-up		47·50 [43·00, 51·00]	46·00 [40·50, 51·00]				
	Change from Baseline		4·00 [0·00, 7·00]	2·00 [0·25, 4·75]	102	−0·7, (−2·45, 1·06)	0·8642	0·000
Falls Efficacy	Baseline		18·48 (13·07)	18·57 (14·13)				
	Follow-up		15·88 (7·99)	17·68 (14·82)				
	Change from Baseline		−2·60 (13·22)	−0·89 (18·20)	105	1·78, (−2·61, 6·18)	0·8642	0·000
PROMIS Physical Health	Baseline		43·89 (7·50)	43·38 (6·59)				
	Follow-up		45·68 (7·38)	45·31 (7·95)				
	Change from Baseline		1·79 (6·45)	1·91 (6·86)	105	2·1, (−0·15, 4·34)	0·4697	0·012
PROMIS Mental Health	Baseline		50·42 (8·48)	49·86 (7·30)				
	Follow-up		49·52 (7·76)	51·22 (8·04)				
	Change from Baseline		−0·90 (5·86)	1·36 (5·95)	104	−0·06, (−2·7, 2·57)	0·9610	0·021
Average Daily Step Counts	Baseline		3,819 (2,402)	3,699 (1,810)				
	Follow-up		3,330 (1,811)	4,195 (2,108)				
	Change from Baseline		−488 (1,354)	+496 (1,313)	87	985, (427, 1543)	0·0100	---

1 Total number of participants enrolled from 12 clusters (HIW = 7 clusters, CSW = 5 clusters)

2· Secondary outcome adjusted p-values using Hochberg’s approach

3 ICC from mixed model with random center effect

**Fig 2:**
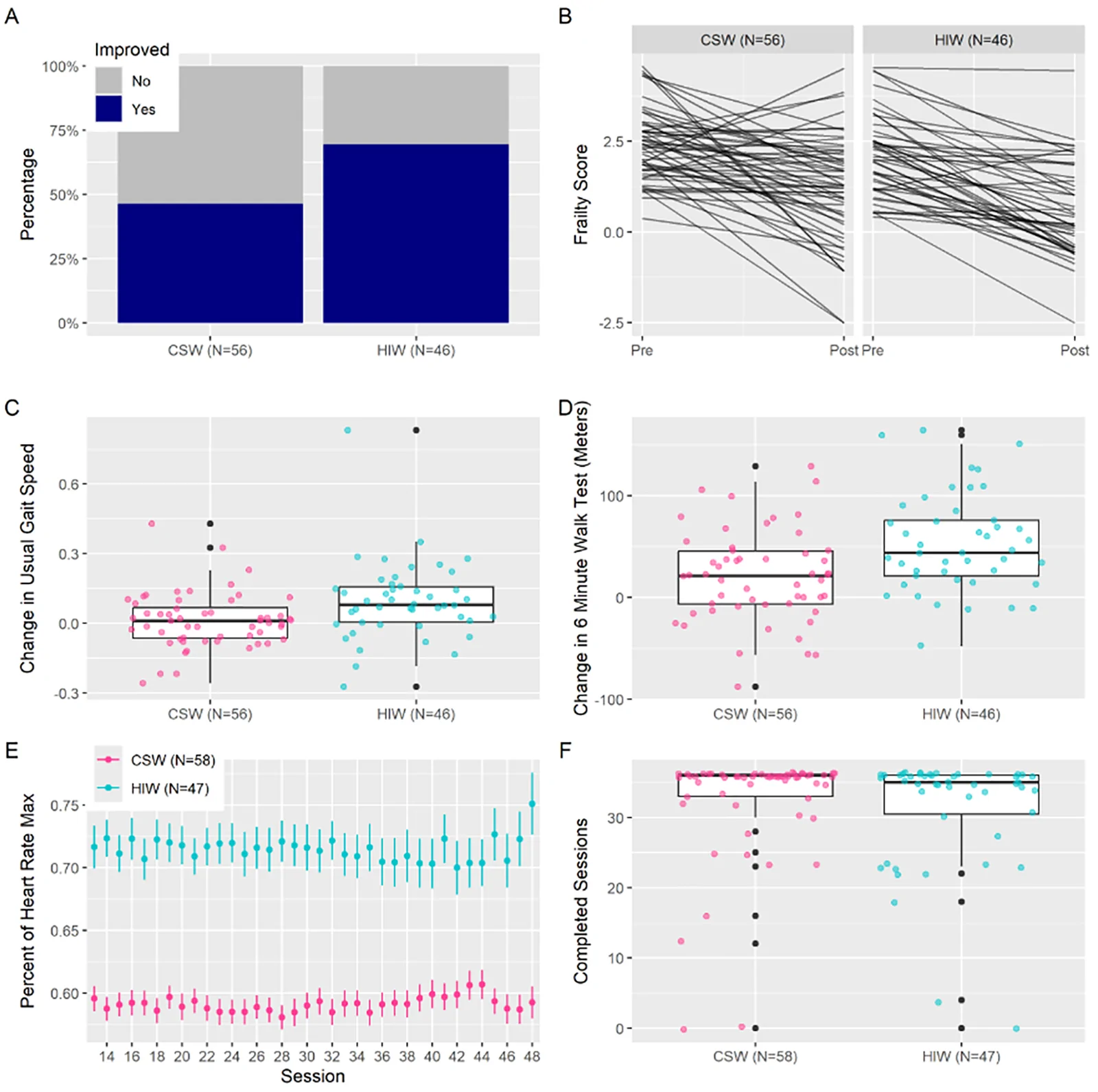
Study Outcomes A: Percent of participants by group with > 1 category improvement in frailty. B: Scatterplot of frailty continuous scores. C: Change in usual gait speed. D: Change in 6 Minute Walk Test. E: Mean percent HR during training sessions. F: Completed sessions.

Sensitivity analyses under various missing data assumptions are shown in S3 Table 5 and Table 6 in S3 File. The most conservative sensitivity analyses assuming all participants lost to follow-up resulted in an attenuated odds ratio for improving at least one SHARE-FI frailty category (OR=1.51 [reference group = CSW], 95% CI 0.72, 3.13, p = 0.27). Analyses based on multiple imputations assuming data were missing at random resulted in a pooled odds ratio estimate of 1.66 (95% CI 0.77, 3.59, p = 0.19). Analyses based on multiple imputations assuming data were missing at random, using the delta method, results in non-significant pooled odds ratios varying from 1.58 to 1.66.

### Secondary outcomes

Results of secondary outcomes are shown in Table 2. Using a continuous frailty score, HIW participants had a mean change of −1.51 (SD: 1.18) points compared with −1.19 (SD: 1.66) points in the CSW group, although this difference was not statistically significant (Fig 2B). There was a significant difference in change in usual gait speed among HIW participants (mean difference = 0.08, 95% CI = 0.03, 0.13, Hochberg adjusted P-value = 0.036) (Fig 2C), as well as 6-Minute Walk Test (mean difference = 48.99m, 95% CI = 21.1, 76.87, Hochberg adjusted P-value = 0.0088) (Fig 2D). There was a significant between-group difference in physical activity as measured by activPAL mean steps per day (mean difference = 985 steps/day, 95% CI = 427, 1543, Hochberg adjusted P-value = 0.01). The per-protocol analysis yielded consistent results identifying significant differences in usual gait speed (Mean difference = 0.08 meters/sec (0.02, 0.14), unadjusted p-value = 0.01) and 6 Minute Walk Test (Mean difference = 50.93 meters (16.10, 85.75, unadjusted p-value = 0.005) favoring the high-intensity intervention. While not significant between groups, the change in fast gait speed and SPPB between baseline and follow up in both treatment groups was greater than the MCID of 0.05 m/s for gait speed [23] and 1.0 for the SPPB [24]. There were no significant differences in any other secondary outcomes.

### Treatment fidelity

Participants in the HIW group averaged 71% of HR_max_ during walking sessions while those in the CSW group averaged 59% of HR_max_ (p < 0.001) (Fig 2E). Median number of sessions completed in the HIW group was 35 and was 36 in the CSW group (Fig 2F). Eighty-five participants (81%) completed at least 4 out of 7 tasks for at least 29 of the 36 sessions (CSW n = 49 (85%), HIW n = 36 (77%)) and were included in the per-protocol analyses.

### Adverse events

There was one definitely related adverse event in the HIW group; a non-injurious fall.

There were 13 serious adverse events (9 in CSW, 4 in HIW); all unrelated to the intervention. There were 2 probably (2 in CSW) and 7 possibly (3 in CSW, 4 in HIW) related adverse events. Probably related events were: one instance of knee pain and one instance of lower leg cramping that began during a session and caused the participants to cancel two sessions. Possibility related adverse events were participant reports of increased back or knee pain that limited participation in sessions for one week.

## Discussion

This is the first study to demonstrate that HIW can reverse frailty in older adults with frailty. After 16 weeks, HIW participants had significantly higher odds of improving at least one SHARE-FI frailty category compared to CSW participants. Numerous prior studies have investigated physical activity, multicomponent training, resistance exercise, and nutrition^3,9^ to improve capacity and physical function, rather than directly modifying the frailty syndrome. In a systematic review of 26 PA intervention studies for frailty, only one study used an aerobic intervention, and this study did not measure frailty as a primary outcome. [25]. In studies that have used frailty as a primary outcome, minimal benefit has been found. Huang et. al, found a 0.02 reduction in frailty index after 26 weeks of training twice-weekly at 70% of heart rate reserve, which did not meet the 0.03 threshold for clinical meaningful change in frailty index scores [26,27]. The combination of aerobic and resistive aerobic activities in our approach may have provided sufficient neuromuscular stimulus by increasing frequency and intensity of activity, despite a shorter intervention duration, to drive improvements in frailty category. Our study advances evidence for the optimal dose of therapeutic interventions for the frailty and provides clear evidence on the substantial benefits of high-intensity walking to decrease and reverse frailty among those with existing frailty. Our approach, which incorporated some resistance elements into aerobic training, was distinct.

### Mobility

In addition to reducing frailty, HIW improved gait speed and 6-minute walk distance. Previous work in both healthy older adults and those with neurologic impairment shows greater amounts of walking practice at higher intensities is associated with superior gains in walking performance [28]. Both treatment groups in our study increased walking volume, but HIW participants achieved greater mobility gains suggesting intensity may be the critical parameter to facilitate mobility improvements. Both treatment groups improved fast gait speed and SPPB in excess of 50% above the MCID threshold with the HIW group having a larger change from baseline in both measures.

### Physical activity volume

Beyond mobility improvements, HIW produced substantial improvements in daily physical activity (PA) compared to CSW. Evidence on whether PA programs can modify daily walking behavior is mixed. Some studies report people compensate for exercise participation by reducing ambulatory PA [29]. while others find no change in ambulatory PA after participating in structured exercise. According to the COM-B model of behavior change [30]. physical capacity is a key determinant of activity behavior. Prior research finds low physical capability restricts participation in daily life events among frail older adults. Further, older adults with frailty average three minutes per week in moderate-vigorous physical activity, 85% of waking hours are spent in sedentary behavior (i.e., sitting), and average daily walking is less than 2,000 steps per day [31]. Thus, improving capability may contribute to improved PA participation and greater amounts of daily walking. We found HIW participants increased PA volume by 496 steps per day from baseline. Wright et. al., reported that the median effect of physical activity interventions on older adults was estimated to be 398 steps/day [32]. Our 985-step between group difference places this effect over the 95th percentile based on seven meta-analyses of 160 trials with over 73,000 older adults [33]. These findings suggest HIW meaningfully improves capability and transfers into greater daily activity which is one of the first findings of the benefits of HIW transfer to post-intervention ambulatory PA.

### Physical function

HIW participants also improved their physical function. The change in SPPB score among HIW participants is higher than the change seen in both the REACT trial [33] and the LIFE study [34]. A chief difference between those studies and our approach is intensity of the intervention with low to moderate intensities in REACT and LIFE. Our finding suggests that older adults with frailty may require a more intense intervention to elicit functional improvements. As aging is associated with reductions in aerobic capacity, cardiac output, and other measures of cardiovascular health, lower intensity training may not provide a sufficient physiologic stimulus to induct meaningful cardiovascular or metabolic adaptions. In contrast, HIW likely produces greater cardiorespiratory demand, resulting in increased physiologic reserve and physical function.

### Safety and adherence

A common criticism of high intensity training for older adults with frailty is safety and injury risk. [35]. Across 3,354 sessions in this trial, 0.3% of sessions were associated with an adverse event providing strong evidence that HIW is safe for those with frailty. Another concern of HIW is that participants will be less adherent to more intensive activity [36]. Adherence between treatment arms was similar: HIW at 73% and CSW at 75% refuting concerns that older adults cannot sustain higher-intensity activity. Importantly, participants self-selected their pace and this approach produced clear heart rate differences between groups which achieved targeted intensities. Others have found that self-selecting intensity level during exercise rather than having it imposed may foster greater tolerance to higher intensity levels [37]. Our study provides evidence that older adults can self-select a speed that can achieve more intensive exercise. As many exercise guidelines instruct people to achieve “moderate to vigorous” levels of activity, it may be that a lay-friendly instruction such as “walk as fast as you can” is more easily understood by older adults and able to be acted upon.

### Contributions to research

Our study has four major strengths. First, the intervention was conducted in retirement communities demonstrating the real-world practicality of this approach. Second, this intervention was supervised with scripted, standardized prompts, reducing the likelihood of a research assistant-effect on outcomes. Third, we compared two active interventions, both of which were beneficial, avoiding the now scientifically and ethically questionable no-exercise comparator. Finally, our rigorous design addressed common limitations in frailty research by 1) using frailty as both an inclusion criterion and an intervention outcome via a validated frailty tool, 2) masking both assessors and participants to knowledge of treatment allocation, 3) leveraging the same scripts to motivate participants across groups to minimize the research assistant effect on participants, and 4) directly comparing two exercise intensities.

### Limitations

Although enrollment mirrored the demographics of senior living residents, 75% of our participants were white and 89% were female. Thus, generalizability of our findings to diverse populations needs further investigation. Although this study focused on an exercise-based intervention, frailty is widely recognized as a multidimensional geriatric syndrome influenced by multiple interacting factors. While exercise remains one of the most consistently supported interventions for improving physical function and frailty-related outcomes, multidomain approaches incorporating nutrition, cognitive engagement, and comprehensive geriatric management may provide additional benefit. Future research should explore integrated interventions that address these broader contributors to frailty. One factor that potentially drives the strong adherence to the protocol in this study could be the supervised nature of the program. Further work should explore if the protocol could be delivered and achieve similar results in unsupervised or group settings. We acknowledge a large drop-out rate from enrollment to analysis (36%). Attrition is a widespread challenge in exercise programs and an estimated 50% of participants drop out of exercise programs in the first 6 months [38]. We found the baseline characteristics of the full enrollment sample and the analytic sample were not substantively different. While sensitivity analyses using imputed data did not preserve the significant treatment effect, the magnitude of the effect remained clinically significant. Finally, we did not capture any longer-term outcomes from the intervention or any co-interventions (ex. Physical therapy or structured group exercise participation). Future work should ascertain the impact of co-interventions as well as the impact on longer-term outcomes.

## Conclusion

This study is one of the first investigations in retirement communities targeting changes in physical frailty classification among the pre-frail and frail. This study was robustly designed with participants and assessors masked to group assignment, had good intervention adherence, and was feasible and safe among a vulnerable group of retirement community residents. Contrary to the view that exercise can counteract frailty-related physical impairments but not frailty itself, our findings show that frailty category can be reversed in older adults through a three time per week, 45-minute, 16 week walking intervention that stressed walking as fast as one can and resulted in approximately 70% HR_max_. Our findings suggest increasing intervention intensity is a critical exercise parameter for those delivering exercise programs in clinical practice or in community-based interventions.

## Supporting information

S1 Checklist Study CONSORT checklist. (DOCX)

S2 protocol Study Protocol. (DOCX)

S3 File Intervention Description and Supplemental Analysis including Tables S1-S7. (DOCX)
