# Supplementary material for: A high intensity vs. casual speed walking intervention to reverse frailty among older adults: a cluster randomized controlled trial
Source: PLoS One. 2026 Sep 30;21(9):e0357605. doi: 10.1371/journal.pone.0357605 (PMC13626343; doi:10.1371/journal.pone.0357605)
Supplement: Supplementary file 2 [file pone.0357605.s002.docx]

**Original Trial Protocol:**

**The Effect of High Intensity Walking on Frailty**

**Principal Investigator:**

**Margaret Danilovich, PhD, PT**

**Supported by:**

**The National Institute on Aging**

**AG060162**

**Study Design:**

We will conduct a multi-site, masked-evaluator cluster-randomized controlled intervention trial to investigate the effects of walking intensity on frailty (Aim 1) and mobility, physical functioning, balance, and daily PA assessed during a one-week ambulatory monitoring period (Aim 2). We will randomize 10 retirement communities with 20 older adults at each site to either a: 1) high intensity (HIW) or 2) casual speed (CSW) walking intervention. We define CSW as walking at a self-selected, casual intensity. An example of a self-selected speed walking prescription is evidenced in the National Institute on Aging’s (NIA) Go-4-Life walking recommendation(1) of 30 minutes of daily walking without identifying a specific intensity of walking.

At baseline, we will measure frailty, mobility, physical functioning, balance, and daily PA using an ActivPal activity monitor worn during a one-week ambulatory monitoring period. All participants will receive 3 sessions per week of walking for 4 months for a total of 48 sessions. We have selected 3 sessions per week following the ACSM recommendation for aerobic exercise.(2) We will make every effort to reschedule any missed session during the week of the missed session. However, in the event that we cannot reschedule a session, we will schedule missed sessions during weeks 17-18. We will assess all measures following completion of the 48 sessions and all participants will again wear an ActivPal for a one-week period to assess PA.

**Figure 1:** Fried Model of the Cycle of Frailty

**Rationale for HIW:**

There is robust evidence that HIW improves physical function among populations with neurologic disease as well as healthy younger and older adults. The **central hypothesis** underlying our work is that HIW can have these same benefits among frail older adults. We hypothesize that HIW can reduce or reverse frailty by improving walking speed, increasing PA, enhancing strength and balance, and reducing disability (**Figure 1**). Our pilot data provides evidence for the hypothesis that HIW significantly improves walking speed and results in reduced frailty.

**Rationale for CSW:**

Given the widespread knowledge of the benefits of PA, it would be unethical to have a no-treatment control group. We have 3 reasons for using a self-selected, casual speed intervention as the comparator group: 1) a self-selected speed is what NIA Go-4-Life Walking for Your Health materials recommend, 2) clinicians working with older adults have traditionally used general conditioning activities focused on self-selected, casual speed walking,(3) and 3) studies of walking programs with retirement community residents commonly utilize self-selected, casual speed interventions.(4, 5) Because we ultimately aim to make our findings scalable across all retirement communities nationwide, selecting a comparator group that most closely resembles “usual care” will allow us to compare the effectiveness of these two treatment approaches. Further, our findings will also provide information on the degree to which self-selected, casual speed walking invokes health gains and reduces frailty.

**A.1 Preliminary Study**

We tested a HIW intervention with five pre-frail and frail older adult assisted living residents aged 80+ years.(6) Participants (average age 87 years) received 12, 30-minute HIW sessions over 5 weeks. Between baseline and a 6-week follow-up, we found a very large (2.4 point) reduction in frailty as measured on the Survey of Health, Ageing and Retirement in Europe-Frailty Instrument (SHARE-FI) (Fig 2A) **(see next page)**, a valid frailty measure designed for primary care settings(7) based on the Fried frailty phenotype. We also found significant improvements in self-selected gait speed (Fig 2B), Berg Balance Scale (Fig 2C), and the 6-Minute Walk Test (6MWT) (Fig 2D). **Most importantly, the intervention elicited marked improvements in frailty category in all participants.** Three participants improved from frail to pre-frail categories on the SHARE-FI, one participant progressed from pre-frail to non-frail, and another reversed frailty from frail to non-frail after only 5 weeks of intervention. Differences in gait speed, Berg Balance, and 6MWT were above the minimally clinical important difference (MCID) for older adults emphasizing the clinical significance of these data. Our finding of robust balance improvements is particularly notable as balance tasks were not explicitly practiced during walking and were not a specific focus of intervention sessions.

A - Frailty	B – Gait speed	C – Berg Balance	D – 6 Minute Walk

Baseline	Follow-up	Baseline	Follow-up

Baseline	Follow-up	Baseline	Follow-up

**Figure 2.** Changes in SHARE-FI frailty scores (A), gait speed self selected (ss) (B), Berg Balance (C) and 6 Minute Walk Test (6MWT) (D) from baseline to 6 week follow-up. All values p < 0.05. Note: in (C), circular values 1 and 2 represent outlier values for 2 participants. In (D), asterisks represent outlier values for 2 participants.

We used a Modus StepWatch with all participants to obtain ambulatory activity. Participants showed a 17% increase in average daily step count from 1,680±1,060 at baseline to 1,970±860 at post-test suggesting **HIW may reduce frailty and enhance mobility such that older adults have greater capability to walk more and increase PA levels.** Finally, our preliminary data demonstrates the feasibility, acceptability, and safety of this HIW intervention (**Table 1**). Participants did not report any adverse events and there were no falls either during supervised sessions or outside of walking sessions during the study interval.

**Table 1**: Results from preliminary HIW study with pre-frail and frail older adults

| **Construct** | **Results** |
| --- | --- |
| Feasibility | All participants reached targeted heart rate intensity. Average peak Rating of Perceived Exertion (8) was 16.6/20 (hard to very hard). |
| Acceptability | Participants regarded the overall 1:1 supervised intervention as highly satisfactory (9.6/10). |
| Safety | No adverse events (including falls) were reported in or out of walking sessions. |

**Conclusions:** Our preliminary study demonstrates our experience and ability to recruit, retain, and safely engage older adults in a HIW intervention. Our pilot study provides initial evidence for the HIW intervention and documents our ability to conduct training protocols and assess outcomes. **Next Steps:** We now seek to test the effectiveness of HIW compared with a CSW intervention in a fully-powered, cluster randomized study.

**A.2 Methodology**

**A.2.1 Participant Population for both aims:**

Inclusion criteria:

- Pre-frailty or frailty as determined on the SHARE-FI.
- Able to ambulate at least 10 feet with moderate assistance (<50% physical assistance) or less. Participants will be able to use their assistive device (e.g. cane or walker) during sessions.
- Resident of one of the 10 enrolled retirement community locations (see Letters of Support). Individuals must be > 60 years of age to move into the retirement communities by virtue of the individual community policies.
- Provision of informed consent and clearance for participation as determined by the EASY: Exercise and Screening for You.(9)

Exclusion criteria:

- Uncontrolled cardiovascular, metabolic, renal, or respiratory disease that limits exercise participation (e.g., previous heart attack within 3 months of enrollment or uncontrolled diabetes)
- Resting blood pressure > 180/110 mmHg.(10)

We will not exclude participants on the basis of Mini-Mental State Examination (MMSE) scores as long as all other inclusion criteria are met. **This is a community-based clinical trial involving older adult retirement community residents.** The retirement communities enrolled in this study provide meal services to residents and assistance is available for tasks such as bathing, medication management, and housekeeping. To make our findings generalizable and scalable among retirement communities, we aim to enroll pre-frail and frail older adults with a full range of cognitive functioning as long as all other inclusion criteria is met. **Research estimates 20-33% of retirement community dwelling older adults > 71 years of age have cognitive impairment.**(11, 12)^,^ (13) Our letters of support indicate that 10-60% of residents in our enrolled retirement communities have cognitive impairment. For participants with a MMSE score < 24 (cognitively impaired adults), we will seek consent from the individual’s legally authorized representative. Risk to participants is further mitigated as all walking sessions are individually supervised. The Northwestern Institutional Review Board (IRB) has previously approved this protocol and determined physician approval is not needed for participation given that participants are screened using the EASY and that sessions are individually supervised with a research staff member monitoring vital signs. We will not exclude participants should they be hospitalized or ill. We will make every effort to reschedule a missed session during the week of the missed session. In the event that a session cannot be rescheduled, we will make up missed sessions during weeks 17-18.

**A.2.2 Screening procedures:** Participants will undergo an initial screening by the research coordinator to determine the presence of frailty. The purpose of the initial screening is to avoid an unnecessary visit to complete the entire baseline assessment if the interested person is non-frail. The research coordinator will administer the Groningen Frailty Index (GFI) in-person during recruitment times at resident meetings. Persons scoring 4 or more points on the GFI(14) will then be oriented to the study, sign informed consent, and make an in-home appointment with the masked evaluator (Dr. Healey) to complete baseline measurements. Baseline measurements will confirm the presence of pre-frailty or frailty and will rule out any medical conditions that preclude participation in walking. If a participant is determined to be pre-frail or frail on the SHARE-FI(15) and meets all other inclusion criteria, we will proceed with enrollment in the study.

| **Table 2:** Retirement Communities Enrolled in the Proposed Study | | | | |
| --- | --- | --- | --- | --- |
| **Site Name** | **Total Residents** | **Percent Minority Residents** | **Percent Cognitively Impaired Residents** | **Chicagoland Area Location** |
| 1. The Merion | 205 | 9% | 30% | North Suburb |
| 2. Mather Evanston | 310 | 1% | 25% | North Suburb |
| 3. Mather Wilmette | 200 | 1% | 25% | North Suburb |
| 4. Lake Forest Place | 406 | 3% | 25% | North Suburb |
| 5. Luther Village | 2000 | 5% | 10% | Northwest Suburb |
| 6. Gidwitz Place | 125 | 0% | 30% | Northwest Suburb |
| 7. St Pauls House | 130 | 20% | 60% | City of Chicago |
| 8. The Clare | 200 | 5% | 10% | City of Chicago |
| 9. Mills Park Tower | 198 | 55% | 6% | West Suburb |
| 10. Heritage House | 219 | 92% | 10% | West Suburb |

**A.2.3 Recruitment:** Participants will be recruited from the 10 retirement community sites enrolled in this study (see letters of support). All sites are independent living with the exception of 2 assisted living sites (Gidwitz Place and St Pauls House). We will recruit residents through in-person meetings and flyers. Sites in this study **(Table 2)** have been purposefully selected throughout the Chicagoland area to reflect a range of racial/ethnic backgrounds to maximize generalizability of findings.

**A.2.4 Lack of Sex Differences in Response to Treatment:** Despite the differences in frailty prevalence between men and women,(16) there appears to be little difference in how frail men and women respond to PA intervention.(17) Gordon et al.,(18) on the disproportionate prevalence of fraility among females and that between the ages of 65 and 85, there is an increasing gap between sexes such that females have greater co-morbidity and disability compared to males. This may be attributed to higher rates of mortality in men and a ‘survivor’ effect in the oldest-old men such that those who survive to advanced age have the lowest rates of frailty. Despite these differences, PA studies have not revealed differences in treatment outcomes by sex. In the LIFE-P study,^5^ there were no significant differences for the effect of the PA intervention by sex. Given that retirement communities have, on average, a 7:1 ratio of female to male residents,(19) we anticipate enrolling a greater number of female participants. We will make every effort to recruit males so that we are well positioned to conduct exploratory analyses on possible sex differences in response to intervention.

**A.2.5 Participant Retention:** The most important factor in retention of participants in this study is our relationship with these retirement communities, the rapport the PI has with community staff, and the relationship research assistants develop working with participants 3 times per week. Our experience from our pilot showed that residents were highly motivated to participate in walking studies and we had no participant attrition. Further, sites will notify us should residents move or pass away minimizing our loss to follow-up.

**A.2.6 Outcome Measures** (**Table 3**)

| **Table 3: Outcome Measures by Timepoint** | | | |
| --- | --- | --- | --- |
|  | Baseline | Each walking session | Four months |
| **AIM 1** |  |  |  |
| Frailty (SHARE-FI) | X |  | X |
| **AIM 2** |  |  |  |
| Gait speed | X |  | X |
| 6 min walk test | X |  | X |
| TUG | X |  | X |
| SPPB | X |  | X |
| Berg | X |  | X |
| Falls Efficacy | X |  | X |
| Physical activity | X |  | X |
| **Other Data** |  |  |  |
| Demographics | X |  |  |
| GDS | X |  | X |
| MMSE | X |  | X |
| HR, BP, RPE (walking intensity) |  | X |  |
| SAE & AE |  | X |  |
| Attrition |  | X |  |

**Aim 1:** Primary Outcome*: Frailty*

The primary outcome in this study is **frailty** as measured by the Survey of Health, Ageing, and Retirement in Europe-Frailty Instrument (SHARE-FI).^29^ **While the Fried frailty phenotype is the most commonly used frailty measurement tool, it is not an appropriate frailty assessment tool for the retirement community participants in this study.** In the Fried phenotype, physical activity is evaluated using the Minnesota Leisure Time Questionnaire that inquires about frequency of tasks such as mowing the lawn. Retirement community residents do not complete these tasks, not by virtue of their ability, but due to their living arrangement. Using the Fried phenotype would artificially lower physical activity scores, producing invalid and substantially overestimating frailty.

| **Table 4:** SHARE-FI Continous Scores | | |  |
| --- | --- | --- | --- |
| **Frailty Category** | **Females** | **Males** | |
| Non-frail | < 0.315 | < 1.211 | |
| Pre-frail | 0.316 – 2.130 | 1.212 – 3.005 | |
| Frail | > 2.131 | > 3.006 | |

Instead, we will measure the frailty phenotype using the SHARE-FI which assesses fatigue, appetite, weakness, walking difficulties, and low physical activity; constructs from Fried’s frailty phenotype criteria.^2^ Fatigue is measured as a yes/no response to whether the client has too little energy to complete desired tasks. Appetite inquires about the food intake over the last month (diminished, same, or increased). Weakness is assessed by two repetitions of grip dynamometry on each hand. Walking difficulties are assessed by self-report of the ability to walk 100 meters and climb one flight of stairs. Physical activity is measured via self-report of frequency of engagement in low to moderate physical activities. A composite frailty score is generated based on these items and that score is used to classify individuals as non-frail, pre-frail, or frail. Primary outcome for analyses will be a dichotomous outcome, indicating whether an individual moves up at least one ‘category’ of frailty. All participants must meet criteria for pre-frailty or frailty for study inclusion; thus, anyone moving from frail to pre-frail or non-frail or anyone moving from pre-frail to non-frail will be counted as a ‘success’. Those remaining in their baseline frailty categories or increasing in frailty scores will not be counted as successes. We will also look at frailty as a continuous variable (**Table 4**) for secondary analyses, indicating the degree of raw SHARE-FI score change between timepoints. All analyses will control for baseline SHARE-FI scores. We have experience using the SHARE-FI as a frailty measure^65^ with older adult populations and have documented the responsiveness of this tool to interventions.(6, 20)

**Aim 2:**

Primary Outcome: *Mobility, Physical Functioning, Balance, and Physical Activity*

To assess **mobility**, we will measure *gait speed*, *endurance, and overall mobility. Gait speed* will be measured at self-selected (instruction: to “walk at normal comfortable pace”) and fastest-possible speeds (“as fast as you safely can”). *Gait endurance w*ill be tested using the *6-Minute Walk Test* with instruction to complete using the participant’s self-selected speed. *The Timed Up and Go* (TUG) will be used to evaluate overall mobility. Participants will stand from a 17” chair, walk a distance of 10 feet, and return to sitting at their self-selected usual speed. These outcomes are valid, reliable, and sensitive measures related to overall health and mobility, and have been used to predict functional decline and mortality.(21) ^(22)^

To assess **physical functioning**, we will use the *Short Physical Performance Balance (SPPB).* The SPPB is a group of measures comprising gait, static balance, and sit to stand (chair rise) movements to provide an overall assessment of functional status and physical performance.(23)

To assess **balance and fall risk,** we will use the *Berg Balance Scale* and *Falls Efficacy Scale***.** The Berg is a valid and reliable 14-item tool to evaluate static and dynamic balance among older adults.(24) Scores on the Berg range from 0-56 with scores of 0-20 indicating a high fall risk, scores of 21-40 indicating a medium fall risk, and scores of 41-56 indicating a low fall risk. We will use the Falls Efficacy Scale as this is a widely-used measure among older adults that evaluates the fear of falling and balance confidence.(25) In this 10-item questionnaire with scores ranging from 0 to 100, participants rate their confidence in performing a variety of tasks using a 10-point Likert Scale. Scores are totaled and scores of 70-79 indicate a fear of falling while scores of 80-100 indicate a fall risk.

To assess **physical activity,** we will use ActivPal Activity Monitors worn for a one-week ambulatory monitoring period at baseline and 4 months. Since the ActivPal is positioned on the thigh, it will not be affected by walker or cane use. This is a small, lightweight triaxial accelerometer participants wear on the thigh. Data are sampled at 20 Hz in 10 second intervals(26) to record both durations and frequencies of walking, standing, sitting, and lying. We will use total step counts per day as the primary outcome of analysis for Aim 2. We will also explore duration of walking bouts, reductions in sedentary time, and frequency of sedentary to upright transitions both within group and between group. We have significant experience utilizing these approachs for ActivPal activity assessment(27-29) and Dr. Conroy (co-I) will oversee the data analysis.

**Additional Study Variables:**

*Demographics:* Key demographic variables include age, sex, ethnicity, highest level of formal education, and location of residence. Other baseline assessments include the Geriatric Depression Scale (GDS) to assess for depressive symptoms and the Mini-Mental State Examination (MMSE) to assess cognition. The GDS and MMSE will be collected at all assessment timepoints but are not primary outcome variables of interest. These variables may be added in exploratory analyses in order to control for potential confounding effects.

*Walking session parameters:* A key feature of the present protocol is to ensure careful control and assessment of intensity within each walking session. In order to do this, blood pressure, HR, and Borg RPE will be measured at each session. Blood pressure will be measured at rest at the beginning of each session. In addition, we will use Polar heart rate sensors (A370) for continuous HR recordings throughout sessions. All participants will be educated on the Borg RPE scale prior to initiating walking sessions. We will have a paper copy of the scale available for participants to reference at all sessions.

*Safety: Adverse Events (AEs)* are defined as exercise-related discomforts (e.g., muscle and joint soreness/pain), minor injuries (e.g., strains, sprains), and non-injurious falls. *Serious Adverse Events (SAEs)* are defined as hospitalization, surgery, death, injurious falls, or permanent disability. *Attrition* is defined as the number of participants who withdraw from the study or are lost to follow-up. We will track participant attrition at every walking session as well as reasons for attrition.

**A.2.7 Blinding:** Dr. Healey, a board-certified geriatric physical therapist, will collect outcome measures in each retirement community and be masked to group assignment. Only the statistician (Dr. Ciolino) and research coordinator (Laura Diaz) will have a list of participants, sites, and group assignment. All participants will be masked to group assignment. Since randomization will occur at the site level, participants will be unaware of the other treatment arm. The RAs will also be masked to group assignment since RAs leading HIW sessions will only lead sessions for that treatment arm while RAs leading CSW sessions will only lead sessions for that treatment arm to minimize contamination between treatment groups. Study-specific data (e.g., walking session data) that may reveal arm assignment will be restricted in REDCap; the User Rights feature in REDCap allows for restriction such that access to individual data fields can be restricted to just the coordinator, statistician, and the person entering the relevant data to prevent inadvertent unblinding.

**A.2.8 Intervention:** Dr. Danilovich (PI) will train research assistants (RAs) in the walking intervention protocol. Each walking session is an individual session led by a RA. We will not use a treadmill or any other specialized equipment in an effort to make this protocol as general as possible within retirement communities since not all communities have this equipment. All participants will receive 48 walking sessions over the course of the 4- month intervention within hallways and stairwells of their retirement community. All communities have the space to run these sessions safely (**see letters of support**). **Our approach of providing intervention sessions within the retirement community instead of a laboratory setting is a strength of the proposed protocol, allowing us to recruit and enroll the most frail participants for whom community accessibility is limited.** All enrolled participants will receive 3 sessions each week. This 4-month intervention will consist of two phases: (1) a 1-month *ramp-up* phase whereby HIW participants will gradually increase intensity levels to reach 70-80% HRmax and (2) a 3-month *intervention* phase whereby HIW sessions will walk at 70-80% of HRmax. Each walking session lasts approximately 45 minutes of which 15 minutes will be allocated to warm-up and cool-down and 30 minutes to walking. Participants will be able to take rest breaks as necessary and rest breaks do not count towards the 30 minutes of total walking per session. During all training sessions, RAs will measure blood pressure and assess heart rate using Polar A370 continuous heart rate monitors to ensure each participant is walking within the individually calculated heart rate intensity range given the treatment arm assignment to ensure intervention fidelity. We will also use the Rating of Perceived Exertion (RPE) Scale to record self-reported exertion during walking in both treatment arms. **This supervised approach was successfully used in our pilot study and ensures safety of all participants during sessions.**

**A.2.8.1 *High Intensity Walking****:* The primary goal will be to maximize intense walking activity for 30 minutes in addition to a 15 minute warm-up and cool-down with rest breaks as needed at an intensity of 70-80% of the maximum heart rate. The recommendation of 70-80% matches the ACSM and CDC definition of vigorous intensity physical activity.(30, 31) We will document the time during the session in which rest breaks occur and the duration of rest breaks. Rest breaks will not count towards the 30 minutes of total walking training each session. The primary goal will be to perform continuous walking within the targeted 70-80% heart rate zone.	**This is a community-based clinical trial involving older adult retirement community residents.** As such, we will estimate participants HR_max_ instead of requiring participants to come to our research laboratory to perform maximal heart rate testing. There currently exist a number of standardized equations in the literature to estimate HR_max_.(31) In this study, we estimate HR_max_ using the formula: **Age-predicted HR_max_ = 208 – 0.7 x age**.(32) This equation has been shown to better estimate HR_max_ at advanced ages in comparison with the traditional formula where age-predicted HR_max_ = 220-age.^76,^(32) We use the Karvonen formula [Target HR = (HR_max_-HR_rest)_ x %intensity) + HR_rest_](33) to calculate each participant’s target HR zone. Since we do not have a true measure of the participant’s maximum heart rate, the Karvonen formula will provide a more accurate training zone since it accounts for resting heart rate which may be variable among participants.(33) We will first calculate the 70% threshold and then calculate the 80% threshold to obtain a 70-80% HR range using the following formulas:

- 70% target HR = [(HR_max_ - HR_rest_) x (70%)] + HR_rest_
- 80% target HR = [(HR_max_ - HR_rest_) x (80%)] + HR_rest_

Heart rate range will be reduced by 30 beats per minute if participants are taking beta-blockers following the 2015 Standards for Physical Activity and Exercise from the Association of Chartered Physiotherapists in Cardiac Rehabilation.(34) We will also use the RPE scale to supplement HR monitoring and record these values every 5 minutes with the training goal of 15-17 RPE. When leading HIW, RAs supervising walking sessions will select from overground and stair climbing tasks as needed to enable the participant to reach the walking training goal of 70-80% of HRR or RPE 15-17. The HIW sessions will incorporate both overground walking tasks in hallways and stair climbing over static stairs using step-to or reciprocal walking patterns with progression to higher speeds and reduced handrail use as tolerated. Overground training will focus on speed and variable tasks such as walking in multiple directions, over inclines and obstacles, and/or leg weights with assistive device as needed. This protocol is designed to incorporate variable walking tasks to increase intensity as well as simulate walking tasks encountered in the home and community. **If a participant is not reaching his or her targeted HR intensity (i.e. HR is less than the 70-80% range and RPE is <15), the RA will provide verbal encouragement and increase walking intensity to get the participant’s HR and RPE into the appropriate intensity.** If a participant is not able to reach the targeted intensity after 3 sessions, the PI will discuss alternative strategies with the RA to help the participant reach the intensity goal.

**A.2.8.2 *Casual Speed Walking****:* The primary goal will be to walk for 30 continous minutes indoors at the participant’s self-selected, casual pace in retirement community hallways (excluding a 15-minute warm-up and cool-down) with rest breaks as needed . Rest breaks do not count towards the 30 minutes of total walking each session. We are taking a pragmatic approach to this trial in that, at the end of the study, we will calculate the average heart rate intensity that participants self-select to walk after being instructed to walk at a casual pace. We will then test whether there is a difference in intensities between high intensity and casual speed. Participants will be explicitly instructed to self-select a casual speed. If participants reach 70% of HRmax, RAs will remind participants that the goal is a casual, comfortable walking speed. We will record HR and RPE continuously to calculate the intensity at which participants self-select to walk to ensure fidelity of our intervention approach. **Overground and stair climbing tasks will focus on the same exact types of activities as the high intensity training** (i.e. walking in multiple directions, over inclines and obstacles, and/or leg weights with assistive device as needed) while focusing on a self-selected, casual speed.

**A.2.8.5 Adherence and fidelity:** Adherence and fidelity to the randomized intensity level will be monitored through measurement of HR and RPE during each exercise session and recorded by the RA. The fidelity of the intervention will be realized further through analysis of walking session data (i.e. HR, RPE). If a participant is not reaching his or her targeted intensity in the HIW treatment group, the RA will provide verbal encouragement and increase the walking intensity to reach the appropriate intensity.

**A.2.9. Randomization:** Randomization will be performed by Dr. Ciolino (co-I). Retirement communities will be randomized to one of two arms: 1) HIW or 2) CSW via a modified constrained randomization scheme.(35) Since there are 10 total sites to be randomized at a 1:1 ratio, there are 252 possible allocation scenarios given the selection of five sites from the 10 total into arm one with the remaining 5 sites into arm 2 (10 choose 5). Further, the smaller number of sites makes it imperative to control large chance imbalances with respect to key site-level variables that may affect outcomes or serve as surrogates for potential participant-level covariates. The number of residents in a community and the total square footage (e.g. community size) will most likely play important roles in the amount of PA residents accumulate per day, so we plan to control imbalance in resident density across arms (number of residents per square foot). Further, we plan to control imbalance in site-reported percent of minority residents, residents with cognitive impairment, and whether each site is an assisted living or independent living site. To control imbalance in these variables, the modified constrained randomization method will enumerate all 252 possible allocation scenarios and evaluate imbalance in these four variables (density, percent minority, cognitive impairment, type of residence) for each iteration. Those meeting a pre-specified threshold criterion for each variable will be placed in a subset (a pool smaller than 252 with reasonable levels of balance in each variable); the allocation scenario to be implemented will be chosen at random from this smaller subset of acceptable scenarios.

Since all site-level information will be available at the beginning of the study, all sites will be randomized simultaneously. Upon execution of the randomization algorithm specified above, Dr. Ciolino will share the chosen allocation scenario with the research coordinator (Laura Diaz) via a secure electronic folder housed on Northwestern University’s Feinberg School of Medicine’s servers. These two (Dr. Ciolino and Laura Diaz) will be the only study team members with access to the master randomization list. The coordinator (Laura Diaz) will communicate and determine training for the research assistants (RAs) delivering intervention as relevant to their respective sites. Continual review of this data by Dr. Ciolino and Laura Diaz will allow for assessment of adherence to protocol and inform any necessary follow-up training for RAs delivering interventions.

**A.2.10 Statistical Analyses:** We will use descriptive statistics (mean±standard deviation for continuous measures and frequency/percentages for categorical measures) to summarize all baseline data in aggregate as well as across study arms. Analyses will assume parametric modeling assumptions are appropriate (e.g., normality for linear models); in cases of violation of these assumptions, we will explore nonparametric and/or variable transformations. Analyses will be based on the intent-to-treat (ITT) principle, and sensitivity analyses will examine an as-treated dataset according to a set of pre-defined criteria for adherence. Longitudinal analyses will further include fixed time effects and random participant effects to further account for within-participant correlation across time. All quantitative statistical analyses will be performed using SAS software version 9.4 (The SAS Institute Inc, Cary, NC). Residual diagnostics will assess model assumptions and, as appropriate, transformation of variables, non-parametric methods, and/or inclusion of higher-order terms in analytic models may be explored (e.g., interaction, quadratic, cubic, etc.). Analyses will assume a 5% level of significance. If primary outcome analyses are significant at the 5% level, we will employ Hochberg’s procedure to correct for multiple hypothesis tests for all secondary analyses due to the potentially large number of statistical tests. According to this process, we will order all secondary p-values from smallest to largest. We will reject all *m* hypotheses *if p(m)<0.05/m*. Otherwise, we will reject all *(m-1)* hypotheses if *p(m-1)<0.05/(m-1)*.

Power and sample size considerations allow for an approximate 20% attrition rate, an estimate that we deem slightly conservative given the attrition rate in our other work of approximately 17%.(6, 36) In the event of missing data, we will compare baseline characteristics between participants who completed the 4-month assessment to those without 4-month assessment to determine any potential biases leading to differential attrition by group. In the event of larger than expected missing data and/or suspected bias (missing not at random), we will employ multiple imputation strategies in the analysis to handle cases of missing data.

**Specific Analyses by Aim**

**Aim 1**: To test the effect of walking intensity on both frailty category (e.g. frail, prefrail, and nonfrail) and frailty score as a continuous outcome on the SHARE-FI (frailty instrument).

*Hypothesis:* The HIW group will have: *(1A)* a greater percentage of participants who improve one frailty category in comparison to the CSW group and *(1B)* larger changes on SHARE-FI continuous scores at both time points in comparison to the CSW group.

**Analysis:** Primary outcome for analyses is the 4 month classification according to SHARE-FI criteria: whether a participant improved at least one category of frailty classification. This dichotomous outcome will be analyzed via a generalized linear mixed model (GLMM) with fixed arm, baseline SHARE-FI score effects, and random cluster effects (i.e., random site effects). This will allow for distinction between within and between cluster variation and more efficient analyses that will account for intra-cluster correlation (ICC).

**Aim 2**: To test the effect of walking intensity on mobility, physical functioning, balance, fall risk, and total PA.

*Hypothesis:* HIW participants will have: *(2A)* improved mobility as measured by gait speed, Timed Up and Go, and 6 Minute Walk Test, *(2B)* improved physical function on the Short Physical Performance Battery, *(2C)* improved balance as measured by the Berg Balance Scale and Falls Efficacy Scale and *(2D)* greater total PA measured during a 1-week observation period at 4 months in comparison to the HIW group.

**Analysis:** For each dependent measure, the difference between baseline and 4 month scores will be calculated. Generalized linear mixed models will be performed with fixed arm, baseline score effects, and random cluster effects (i.e., random site effects). This will allow for distinction between within and between cluster variation and more efficient analyses that will account for intra-cluster correlation (ICC).

**A.2.11 Sample size rationale**: We will study 10 retirement community sites with 20 participants per site for a total of 200 participants. In our previous work with a similar population of older adults, overall attrition was 17% at 3 months.(36) We now assume 20% attrition since this study will follow participants out to 4 months. Power calculations conservatively assume 20% attrition at both the site and participant level; with 20% attrition at the site level (one site per arm dropout or loss of 40 participants) and 20% participant attrition at remaining sites (16 participants per site), we will have at least 128 participants for whom we will have complete baseline and follow-up data required to compute outcomes on frailty and physical functioning measures at four months. We will report sex differences should they be observed.

For Aim 1, we will use two complementary approaches to compare the impact of HIW and CSW on frailty syndrome. The advantage of using the SHARE-FI to evaluate the primary outcome of frailty is that we can examine the effectiveness of the intervention by categorizing how participants move between frailty categories (frail, pre-frail, and non-frail) and participant change in frailty on a continuum, whereby individuals can reduce frailty within in a given category. This second approach is based on the assumption that any decrease in frailty score is a “success”, even if a person stays within a frailty category, given that one would anticipate frailty to worsen over time in the absence of the intervention. However, our primary focus in this study is to determine if HIW can reduce or reverse frailty more than CSW by shifting participants between categories of frail, pre-frail, and non-frail. For this reason we based our power considerations on the dichotomous outcome using SHARE-FI scores: a decrease in frailty category (e.g., movement from pre-frail to non-frail) will be labeled an improvement or success.

**Figure 3**: Minimum detectable differences between HIW and CSW at various intracluster correlation coefficients

We anticipate the CSW intervention arm to exhibit only small improvements in frailty category; we hypothesize that less than 5% of participants will improve according to our definition based on SHARE-FI category. Since power considerations rely heavily on the control proportion of success, we allowed improvement to range from 1%-5% for all calculations; we further included a calculation assuming 10% improvement to explore the robustness of our sample size in the event of incorrect assumptions. We anticipate an approximate 25%-50% improvement in the HIW arm, corresponding to a difference on the order of 20% to 45% between arms.

| **Table 5:** Power to Detect Meaningful Clinical Differences Between Treatment Arms in Secondary Outcomes | | | |
| --- | --- | --- | --- |
| **Measure** | **Difference** | **SDs** | **Power to Detect Meaningful Difference under Same Assumptions** |
| SHARE-FI | 1.4 points | 0.18 | >95% power |
| Gait speed | 0.10 m/sec | 0.16 | Variable, depending on ICC  (61%-83%) |
| Six-minute walk test | 50 meters | 17 | >95% power |
| Berg | 5 points | 1.4 | >95% power |
| SPPB | 0.5 points | 0.3 | >95% power |
| Falls Efficacy Scale | 10 points | 3.3 | >95% power |
| TUG | 3.4 seconds | 1.38 | >95% power |

With limited knowledge on intra-cluster correlation (ICC), power considerations allowed for varying levels of correlation within cluster. In general, ICC tends to be small in such studies (very seldom as large as 0.05)(37) and **Figure 3** illustrates the minimum detectable percent difference in frailty status under varying scenarios assuming a two-sided 5% type I error rate, 10% type II error rate (i.e., 90% power), four sites per arm, and 16 participants per site on average for analyses. Since we expect between 20% and 45% absolute difference increase in frailty improvement in the HIW arm when compared to the CSW arm, we anticipate adequate power to detect these differences in frailty under almost all foreseeable scenarios with regard to ICC and control proportions. Even if our assumption regarding control proportion was incorrect such that 10% of those in the CSW arm improve, the worst case scenario (ICC = 0.05) will still allow for 90% power to detect a diffence of at least 27% across the two arms. Our minimum expected sample size of 64 participants per arm will also allow us to detect the differences in SHARE-FI, gait speed, 6 minute walk test, Berg, SPPB, Falls Efficacy Scale, and TUG at 16 weeks as illustrated in **Table 5**.

| **Table 6:** Project Timeline Divided in 4-month Increments Per Year | | | | | | | | | | | | |
| --- | --- | --- | --- | --- | --- | --- | --- | --- | --- | --- | --- | --- |
|  | **Year 1 Year Year** | | | **Year 2** | | | **Year 3** | | | **Year 4** | | |
| Training of RAs,  Blinded assessors | X |  |  |  |  |  |  |  |  |  |  |  |
| Recruitment | X | X | X | X | X | X | X | X | X | X |  |  |
| 4 month intervention  Sites 1 & 2 |  | 20 | 20 |  |  |  |  |  |  |  |  |  |
| 4 month intervention  sites 3 & 4 |  |  |  | 20 | 20 |  |  |  |  |  |  |  |
| 4 month intervention  sites 5 & 6 |  |  |  |  |  | 20 | 20 |  |  |  |  |  |
| 4 month intervention  sites 7 & 8 |  |  |  |  |  |  |  | 20 | 20 |  |  |  |
| 4 month intervention  sites 9 & 10 |  |  |  |  |  |  |  |  |  | 20 | 20 |  |
| Data Analyses |  | X | X | X | X | X | X | X | X | X | X | X |
| Dissemination  activities: manuscripts  and presentations |  |  | X | X | X | X | X | X | X | X | X | X |

**A.2.12 Limitations/Benchmarks/Alternative Approaches**

1. *Recruitment feasibility*. All sites are committed to the proposal (**see letters of support**) with sufficient number of residents for recruitment. In our ongoing exercise study with homebound frail older adults (PI: Danilovich),(38) we have enrolled 126 participants in a seven-month period demonstrating our success in working with this frail population. If we are unable to recruit enough participants at each site, we will work with site staff to address alternative recruitment strategies.

2. *Benchmarks for success:* Key benchmarks relate to recruitment, retention, adherence, attrition, and participant outcomes. Achieving full participant accrual will require enrolling 20 participants every 4 months.

3. *Alternative findings and approaches:* There is a possibility our findings will contradict hypotheses. Alternative findings might include: (a) similar improvement in both treatment arms, (b) no reduction in frailty in either treatment arm, or (c) more frailty reduction in the CSW arm. We expect both HIW and CSW to improve due to the nature of an exercise training effect. It is possible that the difference in frailty scores will not reach statistical significance between groups. If this occurs, we will publish the non-significant findings and conduct a power analysis to determine if a larger study would find a statistically significant difference. We will also conduct within-group analysis in order to make recommendations about two equally efficacious treatments. It is possible, though unlikely, that CSW participants will self-select a high walking intensity. However, we will clearly instruct CSW participants to ambulate at a comfortable, casual pace to differentiate the training intensity from HIW.

**A.2.13 Timeline (Table 6):** Achieving full participant accrual will require enrolling and completing 20 participants at each site. We propose to run 2 sites (1 HIW and 1 CSW) simultaneously for 4 months which would allow for recruitment, screening, intervention, and follow-up within each site. Other aspects of the study, including data analysis and dissemination of the results will be conducted continually throughout the study.

**HUMAN SUBJECTS**

**1. Risks to Human Subjects**

**A. Human Subjects Involvement, Characteristics, and Design**

This study will be conducted through Northwestern University Department of Physical Therapy and Human Movement Sciences. Intervention sessions will occur at one of the 10 enrolled retirement communities in the Chicagoland area. Study participants will include older adults with pre-frailty or frailty. All participants will be recruited from one of the 10 enrolled retirement communities with which we have established collaborative relationship. The population studied is from the Chicagoland area, and the data management and protection are described below. We will place recruitment flyers in all resident mailboxes as well as make presentations about the study at resident monthly town-hall meetings. After informed consent is obtained, screening procedures will include the EASY: exercise and screening for you and medical screening question. Participants will be enrolled who meet inclusion criteria and meet no exclusion criteria. We will screen as many participants as necessary to recruit a sample size of 200 participants (20 per site) for the study. Recruitment procedures are detailed below. Retirement communities will be randomized into one of two arms: 1) high intensity walking or 2) casual speed walking. These intervention approaches are based on our extensive experience with walking interventions for pre-frail and frail older adults.

Participant Selection:
Inclusion Criteria:

- > 60 years of age with pre-frailty or frailty as determined on the SHARE-FI
- Able to ambulate at least 10 feet with moderate assistance (<50% physical assistance) or less
- Resident of one of the 10 enrolled retirement community locations (see Letters of Support)
- Able to follow 3-step commands
- Provision of informed consent and clearance for participation as determined by the EASY: Exercise and Screening for You.(9)

Exclusion criteria:

- Uncontrolled cardiovascular, metabolic, or respiratory disease that limits exercise participation (e.g., previous myocardial infarction within 3 months of enrollment, uncontrolled diabetes, end-stage renal disease)
- Resting blood pressure > 180/110 mmHg(10)

Participants may be withdrawn from the study for any of the above criteria and will be referred to the most appropriate medical professional. Serious adverse events (SAEs) will be handled with the standard of Good Clinical Practice Guidelines including informing the PI and IRB within 24 hours of the event, following up and reporting on the event until resolved, and subsequently reported to the Data Safety Monitor.

**B. Sources of Material**

*Screening*

Initial screening of each participant will be held in their own apartment within their retirement community or over the telephone. The research coordinator will initially screen participants to determine frailty. Once determined to be pre-frail or frail and meeting all other inclusion criteria, an appointment will be made with Dr. Healey (masked evaluator) to administer the SHARE-FI screening and completion of all other study measures.

*Sources of Materials*

The following data will be collected on the participants involved in this research study: EASY: exercise and screening for you, Geriatric Depression Scale, Mini-Mental State Examination, SHARE-FI frailty score, gait speed, 6 Minute Walk Test, Short Physical Performance Battery, Berg Balance, Falls Efficacy Scale, and physical activity measured with an ActivPal. We will also capture demographic information such as age, sex, race, and education. All data files will be de-identified and kept in locked files in the study research team office. Only the study biostatistician and research coordinator will have access to individually identifiable private information about human subjects.  All electronic data files housed on the secure network at Northwestern University and will be password protected.  Only investigators and key research personnel will have access to paper and electronic files.

**C. Potential Risks**

We anticipate no psychological, financial, or legal risks to participants in our study. No questionnaires related to psychological status will be used. Participants will be advised to speak with the PI for any issues concerning dissatisfaction with the study. We have piloted our walking intervention with other pre-frail and frail older adults and found no adverse events. Participants may experience muscle soreness due to walking training that will attenuate as expected following a bout of activity. Research assistants will encourage anyone feeling pain to modify the walking activity. Given that this population has multiple chronic conditions, participants will have walking tasks modified as appropriate for their given health conditions and individual limitations. Research assistants will be trained to recognize clinical signs and are able to monitor vital signs. Stethosocopes, blood pressure cuffs, and heart rate monitors will be available at all times. Should participants face any complications, medical staff can be notified. As described in our IRB protocol and consent form, if participants were to receive an injury during the course of the study, the individual would be responsible for their own medical care.

**2. Adequacy of Protection Against Risks**

**A. Recruitment and Informed Consent**

Older adults will be recruited through resident town hall meetings and direct mailing at their retirement community. Participants will be recruited with the combined efforts of the study staff. Dr. Danilovich will be responsible for the main recruitment efforts and will work closely with the assisted living sites and the study coordinator to ensure success. Interested participants will complete SHARE-FI screening to determine eligibility. If eligible (pre-frail or frail), participants will provide informed consent and proceed with all baseline testing. All study documents will be available in English. The consent form and study recruitment materials will explain all procedures, risks, and benefits of the study. These materials will also state that participants have the right to withdraw from the study at any time without giving any reason and without any penalty. Participants will need to communicate their intention to leave the study to any research staff personnel and this confidentiality will not be breached.

**B. Protection Against Risk**

The risks of walking sessions will be minimized by adhering to the following strategies:

*(i)* Personnel training: All research staff will be thoroughly trained in the intervention procedures under the direction of a board-certified geriatric physical therapist. All research assistants will be CPR trained. All research assistants will be instructed in how to implement a special medical call at each community in this study.

*(ii)* Participant screening: All potential participants will complete a medical screening with a board-certified geriatric physical therapist to ensure safety to begin a walking intervention. Serious adverse events are not expected, but all such events will be reported according to the Northwestern IRB policies.

**3. Potential Benefits of Proposed Research to Human Subjects and Others**

Randomized trials of walking training interventions have shown significant improvements in functioning and balance as a result of training. There is potential for participants in the proposed study to experience the same benefits. These outcomes have implications far outweighing the minimal risks such as muscle soreness that are associated with participation.

**4. Importance of the Knowledge to be Gained**

The adverse health consequences of frailty are significant and recognized as a public health burden. Currently, there is a poor understanding of how walking interventions affects frailty in older adults. The proposed study will provide critical new knowledge regarding the impact of a walking intervention on the frailty syndrome in older adults. Individual participants and retirement communities will also gain knowledge on an intervention with health benefits for older adults that can be implemented and sustained within retirement communities. Therefore, the minimal risks to participants are reasonable in relation to the benefits of knowledge relative to physical activity interventions and their role in delaying or reducing frailty to facilitate aging in place.

**5. Data and Safety Monitoring Plan**

All study data will be imported and entered into RedCap using forms and tables. All client data will be de-identified and tracked using a unique identifier. All electronic data will be stored on a dedicated hard drive and password protected. The hard drive is secured in a locked office accessible only to study staff. The password will be changed quarterly and known only by staff directly involved in the study. Hard copy documents will be kept in a locked file cabinet in the study office. All study staff are experts in the maintenance of security and confidentiality of research data. Study staff will review and approve study protocols prior to initiation of enrollment. Dr. Huisingh-Scheetz, the data safety monitor, will review SAEs and AEs quarterly and will be altered to any interim concerns.

Dr. Megan Huisingh-Scheetz from the University of Chicago will oversee safety monitoring. Dr. Huisingh-Scheetz is a geriatrician with expertise in frailty. She is the founding director of the University of Chicago Successful Aging and Frailty Evaluation Clinic in which she evaluates and manages medical care for older adults with frailty. She will provide a mechanism to assure monitoring the overall conduct of the study (including issues with safety, ethics, patient recruitment and accrual, retention, adequacy of the study design to achieve the specific aims) and provide to the study team for the possible protocol amendments, and input from this study on future studies.

**6. ClinicalTrials.gov Requirements**

This study will be registered in the ClinicalTrials.gov per Public law 11/-85/ of the FDA Amendments Act of 2007.

REFERENCES CITED

1.	Aging NIo. Walking for Your Health Available from: <https://go4life.nia.nih.gov/sites/default/files/WalkingForYourHealth_0.pdf>.

2.	American College of Sports Medicine Position Stand. Exercise and physical activity for older adults. Medicine and science in sports and exercise. 1998;30(6):992-1008. Epub 1998/06/13. PubMed PMID: 9624662.

3.	Falvey JR, Mangione KK, Stevens-Lapsley JE. Rethinking Hospital-Associated Deconditioning: Proposed Paradigm Shift. Physical therapy. 2015;95(9):1307-15. Epub 2015/04/25. doi: 10.2522/ptj.20140511. PubMed PMID: 25908526; PMCID: PMC4556957.

4.	Johnson JA, McIlroy WE, Roy E, Papaioannou A, Thabane L, Giangregorio L. Feasibility study of walking for exercise in individuals living in assisted living settings. Journal of geriatric physical therapy (2001). 2013;36(4):175-81. Epub 2013/03/06. doi: 10.1519/JPT.0b013e318282d2d3. PubMed PMID: 23459236; PMCID: PMC5096924.

5.	Taylor L, Whittington F, Hollingsworth C, Ball M, King S, Patterson V, Diwan S, Rosenbloom C, Neel A, Jr. Assessing the effectiveness of a walking program on physical function of residents living in an assisted living facility. Journal of community health nursing. 2003;20(1):15-26. Epub 2003/02/13. doi: 10.1207/s15327655jchn2001_02. PubMed PMID: 12581940.

6.	Danilovich M, Conroy D, Hornby TG. Feasibility and Impact of High Intensity Walking Training in Frail Older Adults. Journal of aging and physical activity. 2017:1-17. Epub 2017/01/26. doi: 10.1123/japa.2016-0305. PubMed PMID: 28120633.

7.	Romero-Ortuno R, O'Shea D, Kenny RA. The SHARE frailty instrument for primary care predicts incident disability in a European population-based sample. Quality in primary care. 2011;19(5):301-9. Epub 2011/12/22. PubMed PMID: 22186172.

8.	Bowen DJ, Kreuter M, Spring B, Cofta-Woerpel L, Linnan L, Weiner D, Bakken S, Kaplan CP, Squiers L, Fabrizio C, Fernandez M. How we design feasibility studies. American journal of preventive medicine. 2009;36(5):452-7. Epub 2009/04/14. doi: 10.1016/j.amepre.2009.02.002. PubMed PMID: 19362699; PMCID: PMC2859314.

9.	Resnick B, Ory MG, Hora K, Rogers ME, Page P, Bolin JN, Lyle RM, Sipe C, Chodzko-Zajko W, Bazzarre TL. A proposal for a new screening paradigm and tool called Exercise Assessment and Screening for You (EASY). Journal of aging and physical activity. 2008;16(2):215-33. Epub 2008/05/17. PubMed PMID: 18483443.

10.	E.P J. American College of Sports Medicine: Guidelines for Exercise Testing and Prescription. Philadelphia: Lippincott, Williams, & Wilkins; 2000.

11.	Nicholas LH, Bynum JP, Iwashyna TJ, Weir DR, Langa KM. Advance directives and nursing home stays associated with less aggressive end-of-life care for patients with severe dementia. Health affairs (Project Hope). 2014;33(4):667-74. Epub 2014/04/09. doi: 10.1377/hlthaff.2013.1258. PubMed PMID: 24711329; PMCID: PMC4159465.

12.	Rodriguez-Sanchez E, Mora-Simon S, Patino-Alonso MC, Garcia-Garcia R, Escribano-Hernandez A, Garcia-Ortiz L, Perea-Bartolome MV, Gomez-Marcos MA. Prevalence of cognitive impairment in individuals aged over 65 in an urban area: DERIVA study. BMC neurology. 2011;11:147. Epub 2011/11/19. doi: 10.1186/1471-2377-11-147. PubMed PMID: 22093337; PMCID: PMC3226440.

13.	Association As. People with Alzheimer’s Disease and Dementia in Assisted Living 2004 Available from: <https://www.alz.org/national/documents/prevalence_alz_assist.pdf>.

14.	Peters LL, Boter H, Buskens E, Slaets JP. Measurement properties of the Groningen Frailty Indicator in home-dwelling and institutionalized elderly people. Journal of the American Medical Directors Association. 2012;13(6):546-51. Epub 2012/05/15. doi: 10.1016/j.jamda.2012.04.007. PubMed PMID: 22579590.

15.	Romero-Ortuno R, Soraghan C. A Frailty Instrument for primary care for those aged 75 years or more: findings from the Survey of Health, Ageing and Retirement in Europe, a longitudinal population-based cohort study (SHARE-FI75+). BMJ open. 2014;4(12):e006645. Epub 2014/12/30. doi: 10.1136/bmjopen-2014-006645. PubMed PMID: 25537787; PMCID: PMC4275665.

16.	Fried LP, Tangen CM, Walston J, Newman AB, Hirsch C, Gottdiener J, Seeman T, Tracy R, Kop WJ, Burke G, McBurnie MA. Frailty in older adults: evidence for a phenotype. The journals of gerontology Series A, Biological sciences and medical sciences. 2001;56(3):M146-56. Epub 2001/03/17. doi: 10.1093/gerona/56.3.m146. PubMed PMID: 11253156.

17.	Cesari M, Vellas B, Hsu FC, Newman AB, Doss H, King AC, Manini TM, Church T, Gill TM, Miller ME, Pahor M. A physical activity intervention to treat the frailty syndrome in older persons-results from the LIFE-P study. The journals of gerontology Series A, Biological sciences and medical sciences. 2015;70(2):216-22. Epub 2014/11/13. doi: 10.1093/gerona/glu099. PubMed PMID: 25387728; PMCID: PMC4311184.

18.	Gordon EH, Peel NM, Samanta M, Theou O, Howlett SE, Hubbard RE. Sex differences in frailty: A systematic review and meta-analysis. Experimental gerontology. 2017;89:30-40. Epub 2017/01/04. doi: 10.1016/j.exger.2016.12.021. PubMed PMID: 28043934.

19.	Communities AS. Assisted LIving Statistics - A Deeper Dive into the Demographics 2016 [cited 2017 September 8th]. Available from: <http://www.ascseniorcare.com/assisted-living-statistics-a-deeper-dive-into-the-demographics/>.

20.	Danilovich MK, Diaz L, Ciolinio JD, Corcos DM. Functional resistance activities to impact frailty: a protocol for a randomized controlled trial involving home care aide and frail older adult dyads. Contemp Clin Trials Comm. 2017;7(September):28-32.

21.	Studenski S, Perera S, Wallace D, Chandler JM, Duncan PW, Rooney E, Fox M, Guralnik JM. Physical performance measures in the clinical setting. Journal of the American Geriatrics Society. 2003;51(3):314-22. Epub 2003/02/18. PubMed PMID: 12588574.

22.	Fritz S, Lusardi M. White paper: "walking speed: the sixth vital sign". Journal of geriatric physical therapy (2001). 2009;32(2):46-9. Epub 2009/12/31. PubMed PMID: 20039582.

23.	Pavasini R, Guralnik J, Brown JC, di Bari M, Cesari M, Landi F, Vaes B, Legrand D, Verghese J, Wang C, Stenholm S, Ferrucci L, Lai JC, Bartes AA, Espaulella J, Ferrer M, Lim JY, Ensrud KE, Cawthon P, Turusheva A, Frolova E, Rolland Y, Lauwers V, Corsonello A, Kirk GD, Ferrari R, Volpato S, Campo G. Short Physical Performance Battery and all-cause mortality: systematic review and meta-analysis. BMC medicine. 2016;14(1):215. Epub 2016/12/23. doi: 10.1186/s12916-016-0763-7. PubMed PMID: 28003033; PMCID: PMC5178082.

24.	Jacome C, Cruz J, Oliveira A, Marques A. Validity, Reliability, and Ability to Identify Fall Status of the Berg Balance Scale, BESTest, Mini-BESTest, and Brief-BESTest in Patients With COPD. Physical therapy. 2016;96(11):1807-15. Epub 2016/11/03. doi: 10.2522/ptj.20150391. PubMed PMID: 27081201.

25.	Hill H, McMeekin P, Parry SW. Does the falls efficacy scale international version measure fear of falling: a reassessment of internal validity using a factor analytic approach. Age and ageing. 2014;43(4):559-62. Epub 2014/06/07. doi: 10.1093/ageing/afu059. PubMed PMID: 24903859.

26.	Edwardson CL WE, Bodicoat, DH, Yates T, Davies MJ, Dunstan DW, Healey GN. Considerations when using the activPAL monitor in field-based research with adult populations. Journal of Sport and Health Science. 2017;6:162-78.

27.	Maher JP, Conroy DE. Daily Life Satisfaction in Older Adults as a Function of (In)Activity. The journals of gerontology Series B, Psychological sciences and social sciences. 2015. Epub 2015/09/26. doi: 10.1093/geronb/gbv086. PubMed PMID: 26405078.

28.	Maher JP, Conroy DE. A dual-process model of older adults' sedentary behavior. Health psychology : official journal of the Division of Health Psychology, American Psychological Association. 2016;35(3):262-72. Epub 2015/12/23. doi: 10.1037/hea0000300. PubMed PMID: 26690644.

29.	Ashour M B, K, Yang, CH, Lagoa, C, Conroy DE, Smyth J, and Lanza S. On the mathematical modeling of human physical activity. Proceedings of the IEEE Multi-conference on Systems and Control; Buenos Aires, Argentina2016. p. 1084-91.

30.	CDC. Target Heart Rate and Estimated Maximum Heart Rate 2015. Available from: <https://www.cdc.gov/physicalactivity/basics/measuring/heartrate.htm>.

31.	Reed JL, Pipe AL. Practical Approaches to Prescribing Physical Activity and Monitoring Exercise Intensity. The Canadian journal of cardiology. 2016;32(4):514-22. Epub 2016/02/22. doi: 10.1016/j.cjca.2015.12.024. PubMed PMID: 26897182.

32.	Tanaka H, Monahan KD, Seals DR. Age-predicted maximal heart rate revisited. Journal of the American College of Cardiology. 2001;37(1):153-6. Epub 2001/01/12. PubMed PMID: 11153730.

33.	Medicine ACoS. ACSM Guidelines for Exercise Testing and Prescription 9th ed. Philadelphia Lippincott Williams & Wilkins; 2013

34.	Rehabilation AoCPiC. Standards for Physical Activity and Exercise. 2015

35.	Ivers NM, Halperin IJ, Barnsley J, Grimshaw JM, Shah BR, Tu K, Upshur R, Zwarenstein M. Allocation techniques for balance at baseline in cluster randomized trials: a methodological review. Trials. 2012;13:120. Epub 2012/08/03. doi: 10.1186/1745-6215-13-120. PubMed PMID: 22853820; PMCID: PMC3503622.

36.	Danilovich MK, Hughes SL, Corcos DM, Marquez DX, Eisenstein AR. Translating Strong for Life Into the Community Care Program. J Appl Gerontol. 2016:733464815625833. doi: 10.1177/0733464815625833. PubMed PMID: 26912729; PMCID: PMC4956585.

37.	Campbell MJ, Donner A, Klar N. Developments in cluster randomized trials and Statistics in Medicine. Statistics in medicine. 2007;26(1):2-19. Epub 2006/12/01. doi: 10.1002/sim.2731. PubMed PMID: 17136746.

38.	Danilovich MK, Diaz L, Ciolinio JD, Corcos DM. Functional resistance activities to impact frailty: A randomized controlled trial involving home care aides and older adult dyads. Contemp Clin Trials Comm. 2017;7:28-32.

**Statistical Analysis Plan**

1. **ANALYSIS OBJECTIVES**
   1.1. To test the effect of walking intensity on both frailty category (i.e., frail, prefrail, and nonfrail) and frailty score as a continuous outcome on the SHARE-FI (frailty instrument). Hypothesis: The HIW group will have: (1A) a greater percentage of participants who improve one frailty category and (1B) larger SHARE-FI continuous score changes at 4 months in comparison to the CSW group
   1.2. To test the effect of walking intensity on mobility, physical function, balance, and total PA. Hypothesis: HIW participants will have: (2A) improved mobility as measured by gait speed, Timed Up and Go, and 6-Minute Walk Test, (2B) improved physical function on the Short Physical Performance Battery, (2C) improved balance as measured by the Berg Balance Scale and Falls Efficacy Scale and (2D) greater total PA measured during a 1-week ambulatory monitoring period at 4 months in comparison to the HIW group.
2. **STUDY DESIGN/SAMPLE**
   2.1. **Study Type**

We propose a cluster randomized controlled trial to evaluate the impact of HIW on frailty in older adults. We will randomize 200 participants from 10 retirement communities (20 per site) to a 1:1 supervised, 4-month overground HIW (70-80% HRmax) or self-selected, casual speed walking intervention (CSW). The CSW intervention is based upon the NIA Go4Life recommendations. The 4-month intervention will consist of two phases: (1) a 1-month ramp-up phase in which HIW participants will gradually increase intensity levels to reach 70-80% HRmax and (2) a 3-month intervention phase whereby HIW participants will walk at 70-80% of HRmax. CSW participants will walk at the self-selected, casual speed for all 4 months. Primary and secondary outcomes will be measured at baseline and 4 months. Our overall hypothesis is that HIW will reduce or reverse frailty in comparison to CSW at 4 months, as well as improve overall mobility, balance, physical functioning, and daily PA.

**2.2. Sample Size**

We will study 10 retirement community sites with 20 participants per site for a total of 200 participants. In our previous work with a similar population of older adults, overall attrition was 17% at 3 months. We now assume 20% attrition since this study will follow participants out to 4 months. Power calculations conservatively assume 20% attrition at both the site and participant level; with 20% attrition at the site level (one site per arm dropout) and 20% participant attrition at remaining sites (16 participants per site), we will have at least 128 participants for whom we will have complete baseline and follow-up data required to compute outcomes on frailty and physical functioning measures at four months.

We anticipate the CSW intervention arm to exhibit only small improvements in frailty category; we hypothesize that less than 5% of participants will improve according to our definition based on SHARE-FI category. Since power considerations rely heavily on the control proportion of success, we allowed improvement to range from 1%-5% for all calculations; we further included a calculation assuming 10% improvement to explore the robustness of our sample size in the event of incorrect assumptions. We anticipate an approximate 25%-50% improvement in the HIW arm, corresponding to a difference on the order of 20% to 45% between arms. With limited knowledge on intra-cluster correlation (ICC), power considerations allowed for varying levels of correlation within cluster. Since we expect between 20% and 45% absolute difference increase in frailty improvement in the HIW arm when compared to the CSW arm, we anticipate adequate power to detect these differences in frailty under almost all foreseeable scenarios with regard to ICC and control proportions. Even if our assumption regarding control proportion was incorrect such that 10% of those in the CSW arm improve, the worst case scenario (ICC = 0.05) will still allow for 90% power to detect a difference of at least 27% across the two arms. Our minimum expected sample size of 64 participants per arm will also allow us to detect the differences in SHARE-FI, gait speed, 6 minute walk test, Berg, SPPB, Falls Efficacy Scale, and TUG at 16 weeks as illustrated in the table below.

| Power to Detect Meaningful Clinical Differences Between Treatment Arms in Secondary Outcomes | | | |
| --- | --- | --- | --- |
| **Measure** | **Difference** | **SDs** | **Power to Detect Meaningful Difference under Same Assumptions** |
| SHARE-FI | 1.4 points | 0.18 | >95% power |
| Gait speed | 0.10 m/sec | 0.16 | Variable, depending on ICC  (61%-83%) |
| Six-minute walk test | 50 meters | 17 | >95% power |
| Berg | 5 points | 1.4 | >95% power |
| SPPB | 0.5 points | 0.3 | >95% power |
| Falls Efficacy Scale | 10 points | 3.3 | >95% power |
| TUG | 3.4 seconds | 1.38 | >95% power |

3. **OUTCOMES**

**Primary**

- Frailty (classified as non-frail, pre-frail, or frail based on SHARE-FI) – a dichotomous outcome will be considered, indicating whether an individual moves up at least one “category” of frailty

**Secondary**

- SHARE-FI (continuous measure)
- Gait speed
- Endurance (6 minute walk test)
- Overall mobility (TUG)
- SPPB
- Berg Balance Scale
- Falls Efficacy Scale
- Physical Activity

4. **STATISTICAL ANALYSIS PLAN**
   4.1. *GENERAL CONSIDERATIONS*

We will use descriptive statistics (mean±standard deviation for continuous measures and frequency/percentages for categorical measures) to summarize all baseline data in aggregate as well as across study arms. Analyses will assume parametric modeling assumptions are appropriate (e.g., normality for linear models); in cases of violation of these assumptions, we will explore nonparametric and/or variable transformations. Analyses will be based on the intent-to-treat (ITT) principle, and sensitivity analyses will examine an as-treated dataset according to a set of pre-defined criteria for adherence. Longitudinal analyses will further include fixed time effects and random participant effects to further account for within-participant correlation across time. All quantitative statistical analyses will be performed using SAS software version 9.4 (The SAS Institute Inc, Cary, NC). Residual diagnostics will assess model assumptions and, as appropriate, transformation of variables, non-parametric methods, and/or inclusion of higher-order terms in analytic models may be explored (e.g., interaction, quadratic, cubic, etc.). Analyses will assume a 5% level of significance. If primary outcome analyses are significant at the 5% level, we will employ Hochberg’s procedure to correct for multiple hypothesis tests for all secondary analyses due to the potentially large number of statistical tests. According to this process, we will order all secondary p-values from smallest to largest. We will reject all m hypotheses if p(m)<0.05/m. Otherwise, we will reject all (m-1) hypotheses if p(m-1)<0.05/(m-1).

4.2. *ANALYTIC STRATEGY*
     4.2.1. *Primary Analyses*

Primary outcome for analyses is the 4 month classification according to SHARE-FI criteria: whether a participant improved at least one category of frailty classification. This dichotomous outcome will be analyzed via a generalized linear mixed model (GLMM) with fixed arm, baseline SHARE-FI score effects, and random cluster effects (i.e., random site effects). This will allow for distinction between within and between cluster variation and more efficient analyses that will account for intra-cluster correlation (ICC).

4.2.2. *Secondary Analyses*

For each dependent measure, the difference between baseline and 4 month scores will be calculated. Generalized linear mixed models will be performed with fixed arm, baseline score effects, and random cluster effects (i.e., random site effects). This will allow for distinction between within and between cluster variation and more efficient analyses that will account for intra-cluster correlation (ICC).

4.3. *MISSING DATA*

In the event of missing data, we will compare baseline characteristics between participants who completed the 4-month assessment to those without 4-month assessment to determine any potential biases leading to differential attrition by group. In the event of larger than expected missing data and/or suspected bias (missing not at random), we will employ multiple imputation strategies in the analysis to handle cases of missing data.

**Amendments to the original trial protocol:**

Due to the COVID-19 pandemic and closure of the recruitment sites in this study, several additions and modifications to the original statistical analytics plan and sample size considerations were made.

Sample Size Considerations

Due to a 13-month pause in enrollment because of COVID-19 when visitors were restricted from entering retirement communities, sample size calculations were re-estimated to determine detectable differences in proportions of improvement with an increased number of sites and fewer participants per site, on average. Data from the ongoing trial was not used to inform sample size re-estimates. Sample sizes of 12 participants per site with 12 total sites provided 90% power with a two-sided type one error rate of 0.05 to detect differences in proportions ranging from 14% to 27%, assuming a range of ICC estimates (0.001 to 0.05) and a range of proportions in the control arm (1% to 10%). After inflation for attrition, our revised target was to enroll 14 retirement communities with approximately 14 participants per community.

Randomization and Masking

Given initial challenges with onboarding retirement communities, we modified our original randomization plan and instead implemented the minimal sufficient balance approach (MSB). The MSB algorithm is a covariate adaptive randomization strategy that allows for incorporation of continuous covariates and uses data from previously randomized sites (and enrolled participants) to inform subsequent arm assignments in an effort to prevent serious imbalance. It was extended to the cluster-randomized setting to incorporate site-level covariates (percent minority residents and percent with cognitive impairment).

Statistical Analysis Plan

Additional analyses were considered to evaluate treatment fidelity. We used a generalized estimating equation approach to assess the difference in HR between arms, accounting for the repeated measures across tasks and sessions, assuming a gaussian distribution, independent working correlation structure, and robust standard errors. A nonparametric Wilcoxon rank sum test was used to compare number of completed sessions between arms.

.
