# Supplementary material for: A high intensity vs. casual speed walking intervention to reverse frailty among older adults: a cluster randomized controlled trial
Source: PLoS One. 2026 Sep 30;21(9):e0357605. doi: 10.1371/journal.pone.0357605 (PMC13626343; doi:10.1371/journal.pone.0357605)
Supplement: Supplementary file 3 [file pone.0357605.s003.docx]

**Supplemental Table of Contents**

**Supplemental Table 1. Intervention Description by Week…………………………………………………….….3**

**Supplemental Table 2. Intervention Description (Week 5-16) by Walking Tasks………………………………4**

**Supplemental Table 3. Standardized Instructions and Motivational Prompts for Intervention…………….…5**

**Supplemental Table 4. Demographics of Included and Excluded Participants……………………………….…..6**

**Supplemental Table 5. Distributions of Changes in Frailty Category……………………………………………9**

**Supplemental Table 6a and b. Subgroup Analysis – Descriptive Summary by Sex and Baseline Frailty Category………………………………………………………………………………………………………...……10**

**Supplementary Table 7. Session Data – ITT analysis………………………………………...……………...……11**

**Supplemental Table 1. Intervention Description by Week**

|  | **Treatment Arm** | |
| --- | --- | --- |
| **Weeks** | **HIW** | **CSW** |
| **1** | Walking at a casual, self-selected pace for 45 minutes. | Walking at a casual, self-selected pace for 45 minutes. |
| **2** | Walking at ~50% HRmax for 40 minutes and 5 minutes of step ups. | Walking at casual, self-selected pace for 40 minutes and 5 minutes of step ups. |
| **3** | Walking at ~60% HRmax for 40 minutes and 5 minutes of step ups. | Walking at casual, self-selected pace for 40 minutes and 5 minutes of step ups. |
| **4** | Walking at ~70% HRmax for 40 minutes and 5 minutes of step ups. | Walking at casual, self-selected pace for 40 minutes and 5 minutes of step ups. |
| **5-16** | Walking intervention at 70-80% HRmax for 45min. | Walking intervention at <60% of HRmax for 45min. |

**Supplemental Table 2. Intervention Description (Week 5-16) by Walking Tasks**

| **Time (min)** | **Walking Tasks** |
| --- | --- |
| 10 | Walking Warm up |
| 2·5 | Step ups with the right leg |
| 1 | Rest break |
| 5 | Walking using their AD* |
| 1 | Rest break |
| 5 | Walking without their AD* |
| 1 | Rest break |
| 2·5 | Step ups with the left leg |
| 1 | Rest break |
| 5 | Walking using their AD* wearing 5lbs. ankle weights |
| 1 | Rest break |
| 5 | Walking in variable directions |
| 1 | Rest break |
| 5 | Walking without their AD* wearing 5lbs. ankle weights |
| 5 | Walking cool down |

*AD: Assistive Device

**Supplemental Table 3. Standardized Instructions and Motivational Prompts for Intervention**

|  | **Instruction and Motivation Prompts** | |
| --- | --- | --- |
| **Task/Time** | HIW | CSW |
| Warm up  (start) | “Walk at a comfortable pace” | |
| (at the start of minute 3 and 6) | “Walk a little faster” | “Walk at a relaxed casual speed” |
| Step  (start) | “I want you to walk up and down this step as fast as you safely can going up with the **right (left)** leg”. | “I want you to walked up and down this step at a relaxed, comfortable pace going up with the **right (left)** leg.” |
| (at the start of minute 1) | “You’re doing great – keep it up - go as fast as you safely can” | “You’re doing great - keep it up - go at a relaxed casual speed” |
| Five-minute walking tasks | “We’re going to walk fast for the next 5 minutes. I want you to walk as fast as you safely can.” | “We’re going to walk at a relaxed, comfortable pace for the next 5 minutes. I want you to walk at a casual speed.” |
| (at the start of minute 2) | You’re doing great | |
| (at the start of minute 3) | You’re two minutes in - keep up the good work. Walk as fast as you safely can. | You’re two minutes in - keep up the good work. Walk at a relaxed, casual speed |
| (at the start of minute 4) | Two minutes left - you can do it – keep going faster | Two minutes left - you can do it – keep going |
| (at the start of minute 5) | You are nearly done | |
| (at the end of minute 5) | Good job. You did that task very well | |
| Cool Down  (start) | “Walk at a comfortable pace” | |
| (at the start of 2·5 minutes) | “You are half way done” | |

**Supplemental Table 4. Demographics of Include and Excluded Participants**

| **Characteristic** | **Overall**, N = 165^1^ | **Excluded**, N = 60^1^ | **Included**, N = 105^1^ | **p-value**^2^ |
| --- | --- | --- | --- | --- |
| **age** |  |  |  | <0·001 |
| Mean (SD) | 81·0 (8·2) | 84·1 (7·7) | 79·2 (8·0) |  |
| **Gender** |  |  |  | >0·9 |
| Female | 130 (79%) | 47 (78%) | 83 (79%) |  |
| Male | 35 (21%) | 13 (22%) | 22 (21%) |  |
| **Race** |  |  |  | 0·8 |
| African-American | 32 (19%) | 10 (17%) | 22 (21%) |  |
| Asian | 2 (1·2%) | 0 (0%) | 2 (1·9%) |  |
| Non-White Hispanic | 2 (1·2%) | 1 (1·7%) | 1 (1·0%) |  |
| Other | 1 (0·6%) | 0 (0%) | 1 (1·0%) |  |
| White | 128 (78%) | 49 (82%) | 79 (75%) |  |
| **Assistive Device** |  |  |  | 0·4 |
| Cane | 44 (27%) | 15 (25%) | 29 (28%) |  |
| None | 53 (32%) | 16 (27%) | 37 (35%) |  |
| Other | 9 (5·5%) | 5 (8·3%) | 4 (3·8%) |  |
| Walker | 59 (36%) | 24 (40%) | 35 (33%) |  |
| **High Blood Pressure** | 122 (74%) | 46 (77%) | 76 (72%) | 0·5 |
| **Diabetes** | 36 (22%) | 12 (20%) | 24 (23%) | 0·7 |
| **Hip Pain** | 55 (33%) | 18 (30%) | 37 (35%) | 0·5 |
| **Knee Pain** | 73 (44%) | 26 (43%) | 47 (45%) | 0·9 |
| **Ankle Pain** | 36 (22%) | 12 (20%) | 24 (23%) | 0·7 |
| **Frailty Score** |  |  |  | 0·045 |
| Mean (SD) | 2·4 (1·0) | 2·6 (1·0) | 2·2 (1·0) |  |
| **Gait Speed - Usual** |  |  |  | 0·031 |
| Mean (SD) | 0·8 (0·2) | 0·7 (0·2) | 0·8 (0·2) |  |
| **Gait Speed - Fast** |  |  |  | 0·035 |
| Mean (SD) | 1·0 (0·3) | 0·9 (0·3) | 1·0 (0·3) |  |
| **6 Minute Walk Test (meters)** |  |  |  | 0·060 |
| Mean (SD) | 811·5 (283·5) | 757·7 (266·1) | 842·2 (289·7) |  |
| **Timed Up and Go (seconds)** |  |  |  | 0·001 |
| Median (IQR) | 16·8 (13·4, 21·4) | 19·6 (14·5, 24·7) | 16·4 (13·1, 19·4) |  |
| **SPPB** |  |  |  | 0·10 |
| Mean (SD) | 6·5 (2·5) | 6·1 (2·2) | 6·7 (2·7) |  |
| **Berg Balance Scale** |  |  |  | 0·054 |
| Mean (SD) | 39·9 (10·3) | 37·9 (10·4) | 41·1 (10·1) |  |
| **Falls Efficacy** |  |  |  | 0·009 |
| Median (IQR) | 16·0 (11·0, 22·0) | 17·5 (13·0, 26·8) | 14·0 (10·0, 19·0) |  |
| Unknown | 4 | 4 | 0 |  |
| **PROMIS Physical Health** |  |  |  | 0·3 |
| Mean (SD) | 43·2 (7·4) | 42·4 (8·1) | 43·7 (7·1) |  |
| Unknown | 4 | 4 | 0 |  |
| **PROMIS Mental Health** |  |  |  | 0·030 |
| Mean (SD) | 49·1 (8·2) | 47·2 (8·3) | 50·2 (7·9) |  |
| Unknown | 4 | 4 | 0 |  |
| ^1^n (%) | | | | |
| ^2^Welch Two Sample t-test; Pearson's Chi-squared test; Fisher's exact test; Wilcoxon rank sum test | | | | |

**Supplemental Table 5. Distributions of Changes in Frailty Category**

|  | **CSW** | | **HIW** | |
| --- | --- | --- | --- | --- |
|  | **Frail**  N = 23 | **Pre-Frail**  N = 33 | **Frail**  N = 20 | **Pre-Frail**  N = 26 |
| **Follow-up** |  |  |  |  |
| Frail | 8 (35%) | 5 (15%) | 4 (20%) | 2 (7·7%) |
| Pre-Frail | **11 (48%)** | 17 (52%) | **9 (45%)** | 8 (31%) |
| Non-Frail | **4 (17%)** | **11 (33%)** | **7 (35%)** | **16 (62%)** |

- CSW: 5 worsened (Pre-Frail -> Frail); 25 did not change categories (8 Frail -> Frail; 17 Pre-Frail -> Pre-Frail)
- HIW: 2 worsened (Pre-Frail -> Frail); 12 did not change categories (4 Frail -> Frail; 8 Pre-Frail -> Frail)

**Supplemental Table 6a. Subgroup Analysis – Descriptive Summary by Sex**

|  |  | Female | | Male | |
| --- | --- | --- | --- | --- | --- |
| Time | level | Control | Treatment | Control | Treatment |
| Baseline | Pre-Frail | 24 (58.5) | 24 (57.1) | 11 (64.7) | 3 (60.0) |
|  | Frail | 17 (41.5) | 18 (42.9) | 6 (35.3) | 2 (40.0) |
| Follow-up | Non-Frail | 6 (15.4) | 22 (53.7) | 9 (52.9) | 1 (20.0) |
|  | Pre-Frail | 20 (51.3) | 14 (34.1) | 8 (47.1) | 3 (60.0) |
|  | Frail | 13 (33.3) | 5 (12.2) | 0 (0.0) | 1 (20.0) |
| Change from Baseline | Improved | 15 (38.5) | 30 (73.2) | 11 (64.7) | 2 (40.0) |

**Supplemental Table 6b. Subgroup Analysis – Descriptive Summary by Baseline Frailty Category**

|  |  | Frail | | Pre-Frail | |
| --- | --- | --- | --- | --- | --- |
| Time | level | Control | Treatment | Control | Treatment |
| Baseline | Pre-Frail |  |  | 35 (100.0) | 27 (100.0) |
|  | Frail | 23 (100.0) | 20 (100.0) |  |  |
| Follow-up | Non-Frail | 4 (17.4) | 7 (35.0) | 11 (33.3) | 16 (61.5) |
|  | Pre-Frail | 11 (47.8) | 9 (45.0) | 17 (51.5) | 8 (30.8) |
|  | Frail | 8 (34.8) | 4 (20.0) | 5 (15.2) | 2 (7.7) |
| Change from Baseline | Improved | 15 (65.2) | 16 (80.0) | 11 (33.3) | 16 (61.5) |

**Supplementary Table 7. Session Data – ITT analysis**
